# Die Bedeutung von Aufgabenprompts zur Förderung von Strategieflexibilität sowie prozeduralem und konzeptuellem Wissen beim Lösen quadratischer Gleichungen

**DOI:** 10.1007/s13138-025-00262-y

**Published:** 2025-08-18

**Authors:** Maurus Küttel, Christian Rüede, Fritz C. Staub

**Affiliations:** 1 Pädagogische Hochschule Luzern, Frohburgstraße 3, 6002 Luzern, Schweiz; 2 Pädagogische Hochschule FHNW, Hofackerstraße 30, 4132 Muttenz, Schweiz; 3 Institut für Erziehungswissenschaft, Universität Zürich, Kantonsschulstraße 3, 8001 Zürich, Schweiz

**Keywords:** Strategische Flexibilität, Quadratische Gleichungen, Aufgabenprompts, Sekundarstufe II, Kognitive Aktivierung, Strategy flexibility, Quadratic equations, Task prompts, Upper secondary, Cognitive activation

## Abstract

Im gymnasialen Mathematikunterricht sollen Schülerinnen und Schüler lernen, Verfahren nicht nur korrekt, sondern auch flexibel anzuwenden, beispielsweise beim Lösen von quadratischen Gleichungen. Es wird angenommen, dass Aufgaben, die gezielt zur Anregung spezifischer kognitiver und metakognitiver Prozesse gestaltet sind, die Entwicklung einer solchen Flexibilität unterstützen. Ziel dieses Beitrages ist es, die Abhängigkeit zwischen dem Leistungszuwachs und dem Aktivierungspotenzial der angebotenen Aufgaben zu untersuchen. Hierfür wurden die Prompts von Aufgaben zum Lösen quadratischer Gleichungen aus einer Stichprobe von 39 Klassen mittels einer Inhaltsanalyse codiert. Parallel dazu wurde der Leistungszuwachs der *N* = 739 Lernenden dieser 39 Klassen während einer Unterrichtseinheit zum regelbasierten Lösen von quadratischen Gleichungen gemessen.

Lineare Regressionsanalysen zeigten, dass (a) die Entwicklung der Strategieflexibilität und des prozeduralen Wissens von der Anzahl der im Unterricht angebotenen Aufgaben abhängt, die zum Vergleich vorgegebener Lösungswege auffordern; (b) die Entwicklung der Strategieflexibilität und des konzeptuellen Wissens von der Anzahl der Aufgaben mit metakognitiv aktivierenden Prompts abhängt; (c) eine hohe Anzahl Aufgaben mit Prompts, die ein Verfahren vorschreiben, die Entwicklung der Strategieflexibilität hemmt. Die Befunde verdeutlichen, dass das Aktivierungspotenzial von Aufgabenprompts einen bedeutsamen Prädiktor für den Leistungszuwachs beim Lösen von Gleichungen darstellt.

**Zusatzmaterial online:**

Zusätzliche Informationen sind in der Online-Version dieses Artikels (10.1007/s13138-025-00262-y) enthalten.

## Abstract (en)

In secondary mathematics education, students are expected to apply procedures not only correctly but also flexibly, for example when solving quadratic equations. Tasks that deliberately stimulate cognitive and metacognitive processes support the development of such flexibility. The aim of this paper is to investigate the relationship between students’ learning gains and the activation potential of the tasks provided. To this end, prompts included in tasks on solving quadratic equations based on a sample of 39 classes. In parallel, the learning gains of *N* = 739 students in these classes were measured over the course of an instructional unit on rule-based solving of quadratic equations.

Linear regression analyses showed that (a) the development of strategy flexibility and procedural knowledge depends on the number of tasks that prompt students to compare worked-out solutions; (b) the development of strategy flexibility and conceptual knowledge depends on the number of tasks with metacognitively activating prompts; and (c) a high number of tasks with prompts that prescribe a specific procedure tend to hinder the development of strategy flexibility. These findings indicate that the activation potential of task prompts is a significant predictor of learning gains in solving quadratic equations.

## Einleitung

Ein zentrales Ziel des gymnasialen Mathematikunterrichtes besteht darin, dass Schülerinnen und Schüler lernen, quadratische Gleichungen korrekt und flexibel zu lösen. Dass insbesondere Strategieflexibilität ein relevantes Lernziel darstellt, zeigen aktuelle Curricula für das Gymnasium: Lernende sollen geeignete Lösungsverfahren wählen, diese bewerten (Kultusministerkonferenz 2012, S. 16 ff.) und Lösungsverfahren *flexibel* anwenden können (Eberle et al. 2015). Das heißt, dass sie unterschiedliche Lösungswege erwägen und Gleichungen in möglichst wenigen und möglichst einfachen Schritten lösen können.

Dass es in der Schulpraxis anspruchsvoll ist, diese Kompetenzen zu entwickeln, zeigen empirische Befunde. Viele Lernende in den Sekundarstufen I und II erreichen grundlegende Ziele des Algebra-Unterrichts nicht (Hoch und Dreyfus 2004; Knospe 2011; Malle 1993; Van Stiphout 2011). Malle (1993) zeigte in der Klagenfurter Interviewstudie auf, dass selbst Akademikerinnen und Akademiker an einfachen algebraischen Umformungen scheitern. Ein weiterer Befund ist, dass viele Lernende Gleichungen nicht flexibel lösen können. Das machten beispielsweise Hämmerle et al. (2018) anhand der Gleichung $x-x\left(3x+4\right)=5-x(3x+4)$ deutlich: Obwohl die Lösung durch die Addition von $-x\left(3x+4\right)$ auf beiden Seiten sofort bestimmt werden kann, multiplizieren gut 90 % der Lernenden an Gymnasial- und Fachmaturitätsschulen (vergleichbar mit dem Abitur) im ersten Schritt die Klammern aus; zudem waren die entsprechenden Resultate mehrheitlich falsch. Es stellt sich die Frage, welche Lerngelegenheiten die Kompetenzentwicklung beim Gleichungslösen unterstützen können.

Im aktuellen mathematikdidaktischen Diskurs gilt die kognitive Aktivierung als eines der wichtigsten Qualitätsmerkmale des Unterrichts, was durch zahlreiche empirische Befunde belegt wird (z. B. Klieme und Baumert 2001; Lipowsky et al. 2009). Dieses Qualitätsmerkmal gilt als erfüllt, wenn die Lernenden zu einer eigenständigen und vertieften Auseinandersetzung mit dem Unterrichtsgegenstand angeregt werden. Von besonderer Bedeutung für die kognitive Aktivierung ist das Aktivierungspotenzial der im Unterricht angebotenen Aufgaben. Ein hohes Potenzial zur kognitiven Aktivierung haben Aufgaben, die anspruchsvolle Denkprozesse erfordern, zum Beispiel Argumentieren, Vergleichen oder Analysieren (Ufer et al. 2015). Zahlreiche Studien deuten darauf hin, dass sich Unterrichtsmaterialien mit einem hohen Potenzial zur kognitiven Aktivierung positiv auf die Leistung der Lernenden auswirken können (Newman et al. 2001, S. 21f.; Resnick et al. 2010, S. 174). Ob und in welchem Umfang der Leistungszuwachs beim regelbasierten Lösen von Gleichungen jedoch von der Häufigkeit von Aufgaben mit einem hohem Aktivierungspotenzial abhängt, ist bislang kaum erforscht. Ein Befund von Star et al. (2015) deutet darauf hin, dass dies für Aufgaben zum Vergleichen ausgearbeiteter Lösungswege der Fall ist. Diesbezüglich wurde Folgendes deutlich: Je mehr Zeit für die Bearbeitung solcher Aufgaben aufgewendet wurde, desto größer war der Leistungszuwachs im Bereich des prozeduralen Wissens.

Mit Blick auf die kognitive Aktivierung der Lernenden ist beim Lösen von Gleichungen bedeutsam, welche Anweisung zusammen mit einer zu lösenden Gleichung angeboten wird (vgl. Abschn. 2.4). Solche Anweisungen werden im Folgenden als *Aufgabenprompts* oder schlicht als *Prompts* bezeichnet. Aufgabenprompts beeinflussen die Aktivierung der Lernenden, weil sie vermitteln, welche kognitiven bzw. metakognitiven Prozesse initiiert werden sollen. Je nach Art der angestoßenen Prozesse ist anzunehmen, dass sich unterschiedliche Leistungszuwächse ergeben. In diesem Beitrag wird folgende Frage untersucht: Inwieweit hängt der Leistungszuwachs von Schülerinnen und Schülern beim regelbasierten Lösen quadratischer Gleichungen davon ab, wie viele Aufgaben mit Prompts unterschiedlichen Aktivierungspotenzials im Unterricht eingesetzt werden.

## Theoretischer Hintergrund

### Aktivierungspotenzial von Aufgaben

Kognitive Aktivierung gilt als zentrales Qualitätsmerkmal von Unterricht. Bislang existiert jedoch keine einheitliche und breit geteilte Konzeptualisierung dieses Konstrukts (Wemmer-Rogh et al. 2024). In der Mathematikdidaktik wird Unterricht dann als kognitiv aktivierend bezeichnet, wenn Lernende zu eigenständigem und elaboriertem Denken auf einem für sie herausfordernden Niveau angeregt werden (Kunter et al. 2011; Leuders und Holzäpfel 2011; Ufer et al. 2015). Für Lehrkräfte sind Aufgaben von zentraler Bedeutung, weil sie das Potenzial haben, kognitive Prozesse gezielt anzuregen. Dieses Potenzial wird als *Potenzial zur kognitiven Aktivierung* bezeichnet (z. B. Kunter und Voss 2011). Spätestens seit den TIMSS-Videostudien (Hiebert et al. 2003; Stigler et al. 1999) gilt das Potenzial der angebotenen Aufgaben zur kognitiven Aktivierung als Qualitätsmerkmal von Unterricht. Ein hohes Potenzial haben Aufgaben, die die Schülerinnen und Schüler zu anspruchsvollen Denkprozessen anregen (Ufer et al. 2015). Der Ländervergleich im Rahmen der TIMSS-Videostudien (Hiebert et al. 2003; Stigler et al. 1999) legte hinsichtlich des Potenzials zur kognitiven Aktivierung erhebliche Unterschiede zwischen den angebotenen Aufgaben offen. Die Befundlage zur Wirkung der kognitiven Aktivierung auf den Lernzuwachs ist nicht eindeutig (Wemmer-Rogh et al. 2024). Es liegen aber Arbeiten vor, die darauf hinweisen, dass Unterschiede im Aktivierungspotenzial von Aufgaben zu Leistungsunterschieden führen können (Hiebert und Grouws 2007; Kunter et al. 2011; Lipowsky et al. 2009; Stein und Lane 1996), während andere Arbeiten diese Abhängigkeit nicht bestätigen (für eine Übersicht siehe Wemmer-Rogh et al. 2024). Die uneindeutige Befundlage zur Wirkung der kognitiven Aktivierung auf den Leistungszuwachs liegt möglicherweise auch an den unscharfen Konzeptualisierungen.

Diesen Studien zufolge besitzen Routineaufgaben, die lediglich zum Ausführen einer bekannten Prozedur auffordern, wenig Potenzial zur kognitiven Aktivierung. Ein hohes Aktivierungspotenzial hingegen zeigen Aufgaben, die anspruchsvolle Denkprozesse anregen; dazu gehören beispielsweise das Argumentieren und Interpretieren, das Lösen von Problemen, das Herstellen von Verknüpfungen oder das Erkennen von Mustern.

Ein weiterer Aspekt der kognitiven Aktivierung besteht in der Anregung metakognitiver Prozesse. Metakognition wird als Wissen über eigene Denkprozesse sowie als Kontrolle über diese verstanden (Hasselhorn und Labuhn 2006). In mehreren theoretischen Konzeptualisierungen wird die Anregung von Metakognition als Bestandteil der kognitiven Aktivierung aufgefasst (Keller et al. 2024). Kognitiv aktivierend sind in diesem Zusammenhang Aufgaben, die Lernende dazu anregen, ihre Denkprozesse zu planen, zu überwachen und zu reflektieren (Widmer et al. 2024). Die Befundlage zur Wirkung von Metakognition auf den Lernerfolg ist auch uneinheitlich, weil zahlreiche andere Faktoren einen Einfluss haben (Hasselhorn und Artelt 2018). Einige Studienergebnisse deuten jedoch auf einen positiven Zusammenhang hin (Cohors-Fresenborg et al. 2010; Hattie 2023; Stillman und Mevarech 2010).

### Strategieflexibilität beim Lösen von Gleichungen

Strategieflexibilität^1^ ist die Fähigkeit, unterschiedliche Lösungsstrategien anzuwenden und zu bewerten, um ein mathematisches Problem mit einer geeigneten Strategie zu lösen (Verschaffel 2023; Verschaffel et al. 2009). Dazu gehören sowohl das *Strategiewissen*^2^ als auch die *Strategienutzung*^3^ (Rittle-Johnson und Star 2007, 2009; Sievert et al. 2022). Zu Ersterem können das Generieren und Erkennen unterschiedlicher Lösungswege sowie das Beurteilen derselbigen hinsichtlich Effizienz gezählt werden. Als Strategienutzung hingegen wird die Fähigkeit bezeichnet, spontan eine effiziente Lösungsstrategie zum Lösen einer Gleichung zu verwenden: „[…] eine Strategie wird dabei als effizienter als eine andere Strategie angesehen, wenn sie weniger Lösungsschritte benötigt und/oder einfachere Lösungsschritte enthält“ (Heinze et al. 2020, S. 15). Beispielsweise wäre $x^{2}+2x+1=0$ in diesem Sinne einfacher mittels $\left(x+1\right)^{2}=0$ zu lösen als durch Einsetzen in die Lösungsformel, weil das Einsetzen und Berechnen mehr Rechenschritte erfordert. Der Effizienzgewinn durch das Faktorisieren entfällt, falls vor dem Faktorisieren zum Quadrat ergänzt werden muss (z. B. bei der Gleichung $x^{2}+2x-3=0$). Zur Beurteilung der Strategieflexibilität wird lediglich die prinzipielle Effizienz der gewählten Strategie berücksichtigt – nicht jedoch, ob alle Lösungsschritte korrekt durchgeführt wurden.

So gesehen sind die Strategien „Ergänzung zum Quadrat“ und „Formel anwenden“ vergleichbar effizient. In der Regel sind zum Lösen durch Ergänzen mehr Schritte erforderlich, dafür sind sie einfacher als das Anwenden der Formel. Deutliche Unterschiede bezüglich der Effizienz sind vor allem beim Lösen von Gleichungen mit spezifischen Eigenschaften feststellbar. Das ist beispielsweise der Fall, wenn beide Seiten einer Gleichung radiziert werden können.

Empirische Befunde weisen darauf hin, dass prozedurales und konzeptuelles Wissen die Entwicklung von Strategieflexibilität unterstützen – und umgekehrt (Schneider et al. 2011). Prozedurales Wissen liegt vor, wenn zur Lösung eines mathematischen Problems bestimmte Handlungssequenzen (z. B. ein Verfahren) *korrekt* ausgeführt werden (Canobi 2009; Rittle-Johnson et al. 2001; Rittle-Johnson und Star 2007, 2009). Beim Gleichungslösen umfasst das prozedurale Wissen die Fähigkeit, Strukturen in Gleichungen zu erkennen und Umformungsschritte durchzuführen, die nötig sind, um die Lösungsmenge zu bestimmen (Rüede et al. 2023). Eine korrekt gelöste Gleichung lässt darauf schließen, dass das entsprechende prozedurale Wissen verfügbar ist. Konzeptuelles Wissen ist in Anlehnung an Baroody und Dowker (2003) das dem Lösen von Gleichungen zugrunde liegende Wissen. Es umfasst die Kenntnis von Konzepten wie beispielsweise der *Äquivalenzumformung* oder der *Lösungsmenge*. Dieses Wissen befähigt dazu, die korrekte Durchführung von Lösungsschritten zu beschreiben und die Korrektheit von Lösungswegen zu begründen (Rüede et al. 2023).

### Förderung des Lösens von Gleichungen

Mehrere Studien kommen zum Schluss, dass sich das Vergleichen von Lösungswegen positiv auf den Leistungszuwachs beim Gleichungslösen auswirken kann. Unter experimentell kontrollierten Bedingungen wurden Effekte auf die Strategienutzung (Rittle-Johnson und Star 2007, 2009; Rittle-Johnson et al. 2012), auf das Strategiewissen und zum Teil auch auf das prozedurale Wissen (Rittle-Johnson und Star 2009) nachgewiesen.

Weiter wurde in einer experimentellen Interventionsstudie mit 39 teilnehmenden Klassen gezeigt, dass eine Weiterbildung^4^ für Lehrkräfte zum Vergleichen von Lösungswegen den Leistungszuwachs der Schülerinnen und Schüler steigert (Rüede et al. 2023). Die erste Experimentalgruppe (EG_Vergl) wurde in der Weiterbildung ins Vergleichen von Lösungswegen eingeführt. Thematisiert wurde, wie die Entwicklung von Strategieflexibilität beim Lösen von quadratischen Gleichungen durch spezifische Aufgaben unterstützt werden kann. Dazu wurden den Teilnehmenden zwölf Aufgaben angeboten, die zum Vergleichen von zwei ausgearbeiteten Lösungswegen anregen (vgl. Abb. 2). Zudem erhielten sie 120 Aufgaben, bei denen es nicht nur um das Lösen der Gleichungen ging, sondern zusätzlich um das Nachdenken über eigene Lösungswege, indem beispielweise ein effizienter Weg gefordert wurde.

**Abb. 1:**
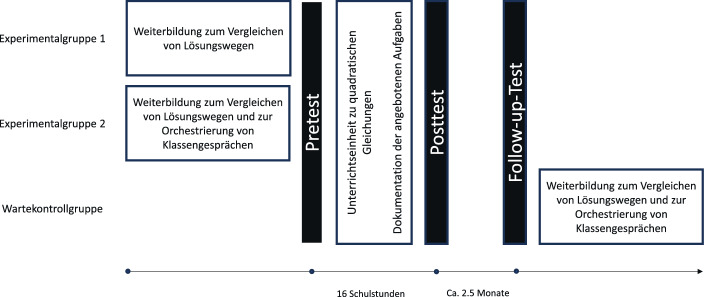
Untersuchungsdesign: Interventionen und Messzeitpunkte (vgl. Rüede et al. 2023)

Die zweite Experimentalgruppe (EG_Vergl & AT) besuchte eine Weiterbildung im gleichen zeitlichen Umfang. Zusätzlich zum Vergleichen wurden die Teilnehmenden dieser Gruppe in den *accountable talk* (Michaels et al. 2013) eingeführt, um das Vergleichen in Klassengesprächen produktiver gestalten zu können. Die Lehrpersonen der Wartekontrollgruppe absolvierten die gleiche Weiterbildung wie die zweite Experimentalgruppe, jedoch erst nach der Unterrichtseinheit und der Datenerhebung (vgl. Abb. 1).

Die Studie zeigt signifikante Haupteffekte der Weiterbildungen auf den Leistungszuwachs der Lernenden. Darüber hinaus arbeiteten Lehrpersonen der Experimentalgruppen häufiger als jene der Kontrollgruppe mit Aufgaben, die zur Auseinandersetzung mit unterschiedlichen Lösungswegen anregten. Allerdings wurde nicht untersucht, ob eine hohe Anzahl an Aufgaben mit einem hohen Aktivierungspotenzial im Hinblick auf multiple Lösungswege zu einem höheren Leistungszuwachs führt. Eine entsprechende Abhängigkeit erscheint auf Grundlage der Ergebnisse der COACTIV-Studie (Baumert et al. 2010) plausibel, auch wenn dort das Potenzial zur kognitiven Aktivierung im Gegensatz zur vorliegenden Studie ohne Berücksichtigung multipler Lösungswege operationalisiert wurde. Es wurde deutlich, dass das Potenzial zur kognitiven Aktivierung der im Unterricht verwendeten Aufgaben den Lernzuwachs positiv beeinflussen kann. Im folgenden Abschnitt wird aufgezeigt, wie dieses Aktivierungspotenzial beim regelbasierten Lösen von Gleichungen durch die Aufgabenprompts beeinflusst wird.

### Aufgaben zum regelbasierten Lösen von Gleichungen

Regelbasiertes Gleichungslösen meint das Lösen von Gleichungen in der symbolischen Darstellung, also mittels Äquivalenzumformungen und Termvereinfachungen. Aufgaben zum regelbasierten Lösen von quadratischen Gleichungen bestehen im Allgemeinen aus einer Gleichung und einem Prompt.

Damit eine Aufgabe eine Lerngelegenheit für Strategieflexibilität darstellt, muss die verwendete Gleichung auf zwei unterschiedlich effiziente Arten gelöst werden können. Das ist beispielsweise bei $\left(x-3\right)\left(x+2\right)=0$ der Fall. Diese Gleichung kann entweder mittels Ausmultiplizieren und anschliessender Nutzung der Lösungsformel für quadratische Gleichungen gelöst werden, oder aber mittels der Nullsetzung der beiden Klammerausdrücke. Der zweitgenannte Weg ist markant effizienter.

Welche kognitiven bzw. metakognitiven Prozesse mit einer Aufgabe angestoßen werden, ist auch vom Prompt einer Aufgabe abhängig. Aufgrund des Prompts kann beurteilt werden, welche Lernprozesse mit einer Aufgabe initiiert werden sollen. Welche Lernprozesse aufgrund eines spezifischen Aufgabenprompts erwartet werden können, wird anhand der Gleichung $\left(x-3\right)\left(x+2\right)=0$ und unterschiedlicher Aufgabenprompts erörtert. Jeder Aufgabenprompt zum regelbasierten Gleichungslösen kann einer der vier folgenden Kategorien zugeordnet werden.

#### Vergleichsprompts

**Abb. 2:**
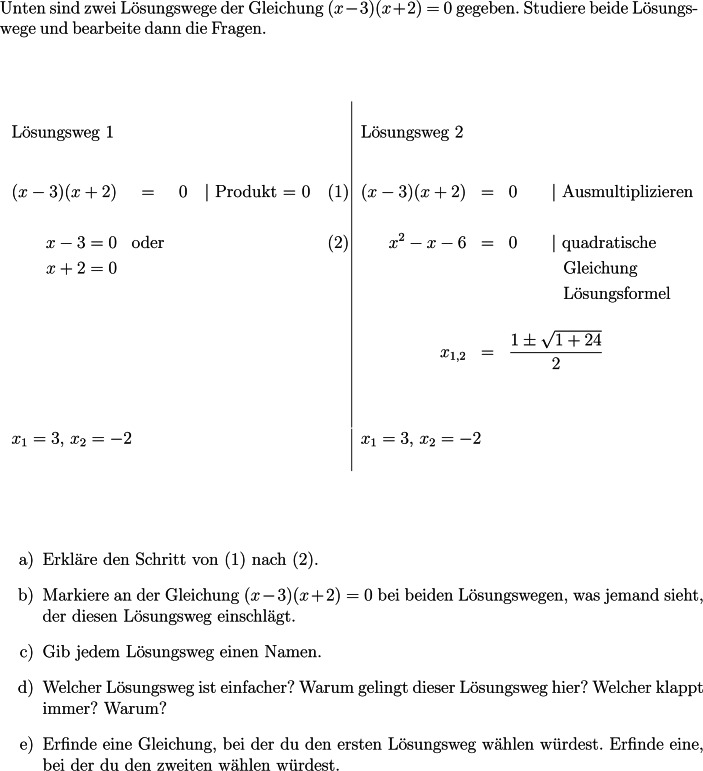
Aufgabe mit Vergleichsprompts (vgl. Rüede et al. 2023)

Vergleichsprompts beziehen sich auf zwei nebeneinandergestellte, *explizit vorgegebene* Lösungswege und fordern dazu auf, Unterschiede und Gemeinsamkeiten festzustellen. Abb. 2 zeigt ein Beispiel für eine Aufgabe mit Vergleichsprompts.

Welche Lernprozesse durch diese Prompts angestoßen werden können, wird anhand der Beispiele (b) und (d) (vgl. Abb. 2) ausgeführt: Teilaufgabe (b) fordert dazu auf, die Gleichung (*x* − 3)(*x* + 2) = 0 genau anzuschauen. Das Potenzial zur kognitiven Aktivierung besteht darin, zu explizieren, wie (*x* − 3)(*x* + 2) = 0 interpretiert werden kann. Dazu ist eine vertiefte Analyse von (*x* − 3)(*x* + 2) = 0 vorzunehmen. Die Kontrastierung der beiden Lösungswege kann die Schülerinnen und Schüler bei diesem Schritt unterstützen und die Identifikation bedeutsamer Merkmale der Gleichung erleichtern. Bei Lösungsweg 2 reicht es anfänglich, sich auf die linke Seite der Gleichung zu konzentrieren und bei (*x* − 3)(*x* + 2) das Ausmultiplizieren zu erkennen. Das ist bei Lösungsweg 1 anders. Hier sind beide Seiten der Gleichung aufeinander zu beziehen, es sind Bezüge zwischen (*x* − 3)(*x* + 2) und 0 herzustellen. Hier ist (*x* − 3)(*x* + 2) nicht nur ein Term, der ausmultipliziert werden kann, sondern ein Produkt, das als Ganzes gleich 0 ist. Solche Analysen haben die Schülerinnen und Schüler mit Färbungen, Umkreisungen etc. explizit zu machen. Das kann ihnen helfen, spezifische Merkmale der Gleichung mit entsprechenden Lösungsansätzen zu verbinden.

Das Potenzial von Teilaufgabe (d) (vgl. Abb. 2) besteht darin, Vor- und Nachteile der beiden Wege zu identifizieren. Der Vorteil von Lösungsweg 1 ist, dass die Lösungsmenge in nur einem Schritt bestimmt werden kann, während Lösungsweg 2 den Vorteil hat, dass er immer funktioniert. Die Schülerinnen und Schüler sollen diskutieren, wann es effizient ist, die Klammern auszumultiplizieren und wann nicht. Bei Gleichungen der Form $\left(x-x_{1}\right)\left(x-x_{2}\right)=0$ ist das Ausmultiplizieren nicht effizient. Der Grund dafür ist, dass das Produkt zweier Faktoren genau dann null ergibt, wenn einer der beiden Faktoren gleich null ist (vgl. Teilaufgabe a). Dieser Weg klappt allerdings nur dann in dieser Weise, wenn auf der einen Seite der Gleichung die Null steht.

Aufgaben mit Vergleichsprompts (vgl. Abb. 2) bieten eine Lerngelegenheit für Strategieflexibilität, da sie Lernende dazu anregen, Vor- und Nachteile unterschiedlicher Lösungswege zu vergleichen und zu bewerten. Zugleich fördern sie konzeptuelles Wissen, indem zentrale mathematische Begriffe – etwa *Ausmultiplizieren* oder *Nullprodukt* – anhand konkreter Beispiele aufgegriffen und erläutert werden können. Für welche Begriffe Lerngelegenheiten bestehen, hängt wesentlich vom Prompt ab. Darüber hinaus können solche Aufgaben auch zur Förderung des prozeduralen Wissens beitragen, da die Auseinandersetzung mit verschiedenen Lösungswegen die korrekte Ausführung von Umformungsschritten unterstützen kann.

#### Metakognitive Prompts zum Lösen quadratischer Gleichungen

Aufgaben mit Prompts, die sowohl zum Lösen einer Gleichung auffordern als auch das Potenzial zur metakognitiven Aktivierung haben, werden im Folgenden kurz als *metakognitive Prompts* bezeichnet. Folgendes Beispiel illustriert, was damit gemeint ist:

$$\text{L{\"o}se die Gleichung}\,\left({x}-3\right)\left({x}+2\right)=0\,\text{m{\"o}glichst effizient}$$

Dieser Aufgabenprompt erfordert eine Analyse der Gleichung. Die Schülerinnen und Schüler müssen idealerweise ihren Weg zuerst planen und anschließend evaluieren. Dazu haben sie wesentliche Merkmale der Gleichung zu erfassen und diese mit ihrem Strategierepertoire in Verbindung zu bringen. Möglicherweise können sie die Effizienz unterschiedlicher Wege aber auch erst dann evaluieren, wenn sie mehrere Lösungswege eingeschlagen und die Gleichung ansatzweise oder vollständig gelöst haben. In jedem Fall verlangt der Prompt aber neben dem Lösen der Gleichung auch eine Auseinandersetzung mit eigenen Lösungswegen und den damit einhergehenden kognitiven Prozessen, indem die Effizienz eines oder mehrerer Wege beurteilt werden muss. Das ist beispielsweise auch der Fall, wenn Lernende ihre Lösungswege untereinander vergleichen.

Aufgaben mit metakognitiven Prompts stellen – analog zu jenen mit Vergleichsprompts – Lerngelegenheiten für Strategieflexibilität sowie für die Erweiterung des prozeduralen und konzeptuellen Wissens dar. Der zentrale Unterschied besteht jedoch darin, dass die Gleichung eigenständig gelöst werden muss und Überlegungen zur Effizienz (z. B. das Markieren verschiedener Sichtweisen) sowie zum konzeptuellen Wissen nicht explizit eingefordert werden.

#### Neutrale und einschränkende Prompts zum Lösen von quadratischen Gleichungen

In derzeit verwendeten Algebralehrmitteln (z. B. Deller et al. 2000; Jankovics 2011) werden auch Aufgaben mit *neutralen* oder *einschränkenden* Prompts angeboten. Neutrale Prompts fordern implizit oder explizit, wie im folgenden Beispiel, zum Lösen einer Gleichung auf:

$$\text{L{\"o}se die Gleichung}\,\left({x}-3\right)\left({x}+2\right)=0$$

Obwohl die Gleichung das Potenzial hätte, zur Lösung unterschiedliche Wege zu erwägen und zu evaluieren, ist das Aktivierungspotenzial der Aufgabe geringer als bei Aufgaben mit Vergleichsprompts bzw. metakognitiven Prompts. Es wird nur verlangt, dass ein Lösungsweg gefunden wird. Eine Auseinandersetzung mit mehreren eigenen Lösungswegen hingegen wird nicht explizit angeregt. Das Aktivierungspotenzial beschränkt sich auf das Bestimmen der Lösungsmenge. Somit sind Aufgaben mit neutralen Prompts vor allem Lerngelegenheiten für prozedurales Wissen.

Einschränkende Prompts schreiben ein konkretes Verfahren vor:

Löse die Gleichung $\left({x}-3\right)\left({x}+2\right)=0$ mit der Lösungsformel.

Diese Prompts werden als *einschränkend* bezeichnet, weil sie die Aktivierung auf ein konkretes Verfahren einschränken. Alternative Lösungswege sind nicht vorgesehen. Wird die Gleichung mit einem derart einschränkenden Prompt angeboten, besteht das Aktivierungspotenzial darin, das geforderte Verfahren korrekt anzuwenden. Entsprechend wird nicht angeregt, einen möglichst effizienten Lösungsweg zu finden. Es ist denkbar, dass Lernende, die viele Aufgaben mit einschränkenden Prompts lösen, eine Tendenz entwickeln, beim Lösen quadratischer Gleichungen „mechanisch“ ein Verfahren anzuwenden, das immer funktioniert (z. B. die Anwendung der Lösungsformel). Eine solche Präferenz für eine Problemlösungsstrategie – die selbst dann zum Zuge kommt, wenn sie im Einzelfall nicht optimal ist – wird als Einstellungseffekt bezeichnet (Blech et al. 2020; Luchins und Luchins 1959).

Diese Beispiele illustrieren, dass bei Aufgaben zum Lösen von Gleichungen das Potenzial zur kognitiven Aktivierung von den Aufgabenprompts abhängt, weil daran erkennbar ist, zu welchen Lernprozessen die Lernenden angeregt werden können. Erhalten die Lernenden Aufgaben mit Vergleichsprompts bzw. mit metakognitiven Prompts, werden sie zur Auseinandersetzung mit multiplen Lösungswegen aufgefordert. Entsprechend ergeben sich Lerngelegenheiten für Strategieflexibilität, prozedurales und konzeptuelles Wissen. Dass das Potenzial zur kognitiven Aktivierung von angebotenen Aufgaben mit dem Leistungszuwachs zusammenhängen kann, zeigt eine Analyse von Aufgaben, die im Rahmen von COACTIV bearbeitet wurden (Baumert et al. 2010). Vor diesem Hintergrund stellt sich die Frage, ob der Leistungszuwachs beim Lösen quadratischer Gleichungen von den Aufgabenprompts abhängt.

### Forschungsfragen und Hypothesen

Zusammengefasst ist das Potenzial zur kognitiven Aktivierung ein Aufgabenmerkmal, das Lernprozesse anregen kann. Ein wichtiger Aspekt dieses Aktivierungspotenzials betrifft die Aufgabenprompts. Aufgaben, die zum Vergleichen vorgegebener Lösungswege auffordern (Vergleichsprompts) und solche, die zur Auseinandersetzung mit eigenen Lösungswegen anregen (metakognitive Prompts), haben das Potenzial, die Entwicklung von Strategieflexibilität, prozeduralem und konzeptuellem Wissen zu unterstützen (vgl. Abschn. 2.4). Aufgaben mit einschränkenden Prompts hingegen können die Entwicklung von Strategieflexibilität hemmen. Aus den bisherigen Erwägungen lässt sich folgende Forschungsfrage ableiten:

*Forschungsfrage: Hängt der Leistungszuwachs von Lernenden in einer Unterrichtssequenz zur Thematik „Lösen von quadratischen Gleichungen“ von der Anzahl angebotener Aufgaben mit (1) Vergleichsprompts, (2) metakognitiven Prompts, (3) neutralen Prompts, (4) einschränkenden Prompts ab?*

#### Hypothese 1

Je mehr Aufgaben mit Vergleichsprompts im Unterricht angeboten werden, desto größer ist der Leistungszuwachs der Schülerinnen und Schüler in den Bereichen (a) Strategieflexibilität, (b) prozedurales Wissen und (c) konzeptuelles Wissen.

#### Hypothese 2

Je mehr Aufgaben mit metakognitiven Prompts im Unterricht angeboten werden, desto größer ist der Leistungszuwachs der Schülerinnen und Schüler (a) in den Bereichen Strategieflexibilität, (b) prozedurales Wissen und (c) konzeptuelles Wissen.

#### Hypothese 3

Je mehr Aufgaben mit neutralen Prompts im Unterricht angeboten werden, desto größer ist der Leistungszuwachs der Schülerinnen und Schüler im Bereich des prozeduralen Wissens.

#### Hypothese 4

Je mehr Aufgaben mit einschränkenden Prompts angeboten werden, desto kleiner ist der Leistungszuwachs in der (a) Strategieflexibilität. Hingegen ist (b) im Bereich des prozeduralen Wissens ein umso höherer Leistungszuwachs zu erwarten, je mehr Aufgaben mit einschränkenden Prompts angeboten werden.

## Methode

Die Daten für die vorliegende Arbeit wurden zwischen 2016 und 2018 im Rahmen einer experimentellen Interventionsstudie erhoben (Rüede et al. 2023). Untersucht wurde die Förderung der Strategieflexibilität beim regelbasierten Lösen von quadratischen Gleichungen. Um die Auswirkungen der Intervention auf den Leistungszuwachs der Schülerinnen und Schüler zu untersuchen, wurden zu drei Zeitpunkten die Leistung gemessen. Erfasst wurde jeweils die Strategieflexibilität, das prozedurale und das konzeptuelle Wissen. Zusätzlich dokumentierten die Lehrkräfte alle im Unterricht angebotenen Aufgaben.

### Stichprobe

An der Studie nahmen 39 Lehrkräfte (38 % Frauen) aus der Deutschschweiz teil, die je eine Klasse der Sekundarstufe II unterrichteten. Von anfänglich 821 Lernenden konnten 82 nicht berücksichtigt werden, weil sie die Einverständniserklärung nicht unterzeichneten bzw. zurückzogen, im Verlauf der Datenerhebung in die Klasse eintraten, diese verließen oder weil sie an allen Messzeitpunkten fehlten. Somit umfasst die Stichprobe für die vorliegende Studie 739 Lernende der Jahrgangsstufe neun oder zehn (im Durchschnitt 15,8 Jahre alt [*SD* = 1,10]; 59 % weiblich; 85 % mit Muttersprache Deutsch). Es handelte sich um elf Klassen an Fach- oder Berufsmaturitätsschulen, deren Abschluss zum Studium an Fachhochschulen berechtigt, sowie um 28 Klassen an Gymnasien. Die Varianz der Leistungszuwächse ist jedoch kaum auf den Schultyp zurückzuführen (*ICC* = 0,09 für die Strategienutzung und für die anderen Skalen gilt *ICC* < 0,03). Die an der Studie beteiligten Lehrkräfte nahmen freiwillig an einer Weiterbildung zum Thema Vergleichen von Lösungswegen in Klassengesprächen teil, die ein integraler Bestandteil des Forschungsprojektes war (vgl. Abschn. 2.3). Sie wurden randomisiert einer der drei Versuchsbedingungen zugeteilt (vgl. Abb. 1).

Das vom Projektteam gesetzte Minimalziel für die Unterrichtseinheit war, das regelbasierte Lösen von quadratischen Gleichungen in sechzehn Schulstunden einzuführen und zu konsolidieren. Mit allen Lehrkräften wurde vereinbart, dass sowohl das Anwenden der Lösungsformel als auch die Wahl eines geeigneten Lösungsweges in dieser Zeit behandelt werden müssen. Abgesehen davon waren die Lehrkräfte frei in der Unterrichtsgestaltung und es gab keine Vorgaben zur Verwendung spezifischer Aufgaben. Im Rahmen der Weiterbildung wurden jedoch zwölf Aufgaben mit Vergleichsprompts und zwei vorgegebenen Lösungswegen (vgl. Abb. 2) sowie 120 Aufgaben mit metakognitiven Prompts zur Verfügung gestellt. Weil Aufgaben mit Vergleichsprompts aus mehreren Teilaufgaben bestehen, die in der Summe mehr Bearbeitungszeit erfordern als Aufgaben mit metakognitiven Prompts, wurden nur zwölf Aufgaben mit Vergleichsprompts angeboten.

Alle 39 Lehrkräfte dokumentierten die im Unterricht angebotenen Aufgaben. Dazu notierten sie diese auf einem Formular oder verwiesen auf Lehrwerke, Unterrichtsskripte und Übungsserien. Vereinzelt wurden auch Fotos von Tafelbildern mit im Klassenverband gelösten Aufgaben eingereicht. Es wurde geprüft, ob die verwendeten Aufgaben für alle sechzehn Unterrichtsstunden dokumentiert wurden. In zwei Fällen war diese Dokumentation unvollständig und die angebotenen Aufgaben mussten mithilfe zweier Schülerhefte und des verwendeten Lehrmittels rekonstruiert werden. Der gesamte Aufgabenkorpus besteht aus 2768 Aufgaben zum regelbasierten Lösen quadratischer Gleichungen.

### Analyse der Aufgaben

Die von den Lehrkräften im Unterricht angebotenen Aufgaben zum regelbasierten Lösen quadratischer Gleichungen wurden in einer *inhaltlich strukturierenden qualitativen Inhaltsanalyse* (Kuckartz 2018, S. 97 ff.) codiert. Aufgrund der Theorie wurde ein Kategoriensystem (vgl. Tab. 1) entwickelt. Auf dieser Grundlage wurden alle im Unterricht angebotenen Aufgaben mit der Analysesoftware MAXQDA (VERBI Software 2021) codiert. Die ermittelte Verteilung der Aufgabenanzahl pro Klasse und Kategorie verdeutlicht, mit welcher Häufigkeit in den einzelnen Klassen Aufgaben angeboten wurden – differenziert nach Prompts mit unterschiedlichem Aktivierungspotenzial inklusive solchen ohne Prompts.

**Tab. 1:** Kategoriensystem zum Codieren der Aufgaben auf Grundlage ihrer Prompts

Aufgabenkategorie		Beschreibung der Aufgabenprompts	Ankerbeispiele
1	*Aufgaben mit Vergleichsprompts*	Aufforderung zum Vergleichen von *worked examples* (vgl. Rittle-Johnson und Star 2007, 2009)	Vgl. Abb. 2
2	*Aufgaben mit metakognitiven Prompts*	Kognitive und metakognitive Aktivierung	
	Effiziente Methode	Aufforderungen zum effizienten Lösen einer Gleichung	„Löse $\left(x+1\right)\left(x-3\right)=0$ möglichst geschickt.“
	Reflexion/Selbsterklärung	Aufforderungen zur Planung, Reflexion oder zur Selbsterklärung eines möglichen Lösungsschrittes	„Gegeben ist die Gleichung $\left(x+3\right)^{2}+2=38$. Was bewirkt das Quadrieren des Ausdrucks $\left(x+3\right)$?“
	Multiple oder alternative Lösungswege	Aufforderung zum Lösen einer Gleichung auf mehreren Lösungswegen oder auf einem alternativen Lösungsweg	„Löse $x^{2}-2x+2=1$ auf zwei unterschiedliche Arten.“
			„Bestimme die Lösungen von $\left(x-1\right)^{2}+1=37$ ohne die Lösungsformel.“
	Im Klassenverband gelöste Gleichung (mit mehreren Lösungswegen)	Aufgaben ohne Prompts, bei denen aus der Unterrichtdokumentation hervorgeht, dass im Klassenverband mehrere Lösungen thematisiert wurden	$\left(x-2\right)^{2}+7=-1$
			Erster Lösungsweg: Vereinfachen und in die pq-Formel einsetzen
			Zweiter Lösungsweg: Die Gleichung hat keine reelle Lösung, weil für jedes x auf der linken Seite eine positive Zahl steht und rechts eine negative
	Peervergleich	Aufforderungen zum Vergleichen und Diskutieren der eigenen Lösung mit jenen von Peers	„Löse die Gleichung $\left(x-1\right)^{2}+1=37$ und vergleiche deinen Lösungsweg mit jenem deiner Banknachbarin.“
3	*Aufgaben mit neutralen Prompts*	Aufforderungen ohne spezifische Angaben zur Lösungsmethode oder Aufgaben, die nur aus einer Gleichung bestehen	
	Ohne expliziten Prompt	Aufgaben, die ausschließlich aus einer Gleichung bestehen	$x^{2}-4x+2=0$
	Lösungsaufforderung	Aufforderung zum Lösen einer Gleichung ohne weitere Angaben	„Löse die Gleichung $x^{2}+4x+7=2$.“
4	*Aufgaben mit einschränkenden Prompts*	Das durchzuführende Verfahren zum Lösen der Gleichung wird festgelegt	
	Substitution	Es wird gefordert, dass die Gleichung durch Substitution gelöst werden soll	„Löse die Gleichung $\left(x-7\right)^{2}+2\left(x-7\right)+1=0$ durch Substitution.“
	Klammern auflösen Nenner eliminieren	Aufforderung zum Auflösen von Klammerausdrücken oder zur Multiplikation mit dem Nenner eines Bruches (ohne Variablen) auf beiden Seiten der Gleichung	„Multipliziere aus: $\left(x+1\right)\left(x-3\right)=2$“ „Löse, indem du zuerst die Nenner der Brüche eliminierst: $\frac{\left(x-1\right)^{2}}{3}-\frac{\left(x-1\right)^{2}}{2}=-1.$“
	Ergänzung zum Quadrat	Aufforderung zum Lösen durch eine Ergänzung zum Quadrat	„Löse die Gleichung $x^{2}-6x+1=0$ durch quadratisches Ergänzen.“
	Faktorisieren	Aufforderung zum Faktorisieren	„Löse die Gleichung $x^{2}-3x-3=0$mit dem Klammeransatz.“
	Lösungsformel	Aufforderung zum Anwenden der Lösungsformel	„Wende die *pq*−Formel an zum Lösen von $x^{2}-6x+1=0$.“
	Radizieren	Aufforderung zum Ziehen der Quadratwurzel	„Löse die Gleichung $\left(x+1\right)^{2}-1=24$durch Radizieren.“
	Im Klassenverband gelöste Gleichung (mit einem Lösungsweg)	Aufgaben ohne Prompts, bei denen aus der Unterrichtdokumentation hervorgeht, dass sie im Klassenverband gelöst wurden, wobei nur ein Weg thematisiert wurde	„$2x^{2}-3x=-2$
			Lösungsweg: Zuerst auf beiden Seiten 2 addieren und anschließend die Koeffizienten in die abc-Formel einsetzen.“

Für die Aufgabenanalyse stellt sich zunächst die Frage nach einer geeigneten Segmentierung des Datenkorpus, um reliabel abgrenzbare Codiereinheiten zu definieren. Weil das Aktivierungspotenzial der analysierten Aufgaben stets an eine quadratische Gleichung gebunden ist, wurden die Codiereinheiten so festgelegt, dass sie genau eine Gleichung umfassten. Ein Teil der Aufgaben enthält darüber hinaus einen oder mehrere Prompts, die präzisieren, welche kognitive Aktivierung beabsichtigt ist. Andere Aufgaben bestehen ausschließlich aus einer Gleichung, wobei implizit dazu aufgefordert wird, die Lösungsmenge zu bestimmen. Die gewählte Segmentierung erwies sich im Rahmen der Gegencodierung von 20 % der Aufgaben als hoch reliabel: Zwischen den Codierenden traten keine Abweichungen hinsichtlich der Segmentierung auf.

Beim Codieren wurde pro Codiereinheit jeweils nur der Code für das höhere Aktivierungspotenzial vergeben – auch dann, wenn sich mehrere Prompts auf eine Gleichung beziehen. Auf diese Weise wird das gesamte mit der Gleichung verbundene Aktivierungspotenzial durch einen Code erfasst. Beispiel:

1. Löse die Gleichung $x^{2}+2x+1=0$.
2. Löse die Gleichung auf einem alternativen Weg. Welcher deiner Wege ist effizienter?

Weil sich beide Prompts auf dieselbe Gleichung beziehen, handelt es sich um eine Codiereinheit.

### Kategorisierung der Aufgaben anhand ihrer Prompts

Die vier Hauptkategorien wurden deduktiv aus der Theorie abgeleitet (vgl. Abschn. 2.4), während die Unterkategorien induktiv gebildet wurden. Aufgrund der Literatur zum Vergleichen von Lösungswegen (z. B. Rittle-Johnson und Star 2007, 2009; Rüede et al. 2023) wurde die Kategorie 1 „Aufgaben mit Vergleichsprompts“ gebildet (vgl. Tab. 1). Aufgaben mit Prompts, die zur metakognitiven Auseinandersetzung mit *eigenen* Lösungswegen auffordern, gehören zur Kategorie 2 „Aufgaben mit metakognitiven Prompts“. Solche ohne Prompts oder mit Prompts, die nur zum Lösen auffordern, bilden die Kategorie 3 „Aufgaben mit neutralen Prompts“. Für Aufgaben mit Prompts, die ein bestimmtes Verfahren vorgeben, wurde die Kategorie 4 „Aufgaben mit einschränkenden Prompts“ festgelegt. Tab. 1 zeigt die Kategorien sowie entsprechende Ankerbeispiele (vgl. Tab. 1).

Das Kategoriensystem (vgl. Tab. 1) wurde vom Erstautor entwickelt, wobei an regelmäßig stattfindenden Sitzungen Rückmeldungen vom Projektteam eingeholt wurden. Die Beschreibungen der Kategorien und entsprechende Ankerbeispiele wurden im Codiermanual festgehalten; um mehrdeutige Fälle zu unterscheiden, wurden Codierregeln formuliert.

Mithilfe des Kategoriensystems (vgl. Tab. 1) wurden alle in den Projektklassen angebotenen Aufgaben codiert. Zu Beginn wurden 20 % der Aufgaben neben dem Erstautor auch von einer eigens dafür geschulten Person gegencodiert. Dabei wurde eine Intercoder-Reliabilität über die vier Hauptkategorien (Brennan und Prediger 1981) von κ = 0,81 erreicht, was einer sehr guten Übereinstimmung entspricht. Im Falle von abweichenden Codierungen fand ein konsensualer Abgleich statt. Die restlichen 80 % der Aufgaben wurden danach ausschließlich vom Erstautor codiert.

### Leistungsmessung

Die Leistungsmessung erfolgte mittels des Paper-Pencil-Tests (vgl. Rüede et al. 2023, Beispiel-Items dieses Leistungstests finden sich im Onlineanhang A). Damit wurden die Leistungen der Lernenden zu drei Zeitpunkten erfasst: vor der Unterrichtseinheit (Pretest), nach der Unterrichtseinheit (Posttest) sowie im Follow-up-Test zweieinhalb Monate nach der Unterrichtseinheit (vgl. Abb. 1). Gemessen wurden die Strategieflexibilität (bestehend aus Strategiewissen und -nutzung) sowie das prozedurale und das konzeptuelle Wissen. Um das prozedurale Wissen zu eruieren, wurden die Schülerinnen und Schüler darauf getestet, ob sie Gleichungen korrekt lösen konnten. Sie hatten zehn Minuten Zeit, um zehn Gleichungen (zwei lineare und acht quadratische) zu lösen. Die Strategienutzung wurde mit denselben Gleichungen gemessen. Diesbezüglich wurde beurteilt, ob der gewählte Weg aus wenigen, effizienten Schritten bestand. So ist es beispielsweise bei der Gleichung $\left(x-1\right)^{2}-2=23$ effizient, wenn auf beiden Seiten 2 addiert und anschließend radiziert wird. Nicht beurteilt wurde hingegen, ob die Schritte korrekt durchgeführt wurden. Das Strategiewissen wurde mit drei Subskalen (Newton et al. 2020) gemessen: Diese bestanden aus Items (a) zum Generieren mehrerer Lösungswege (sechs Items in 6 min), (b) zum Erkennen von unterschiedlichen Lösungswegen (16 Items in 4 min) und (c) zum Bewerten der Effizienz von Strategien (sechs Items in 5 min). Der Teil zur Messung des konzeptuellen Wissens musste in zehn Minuten bearbeitet werden und bestand aus Aufgaben zum Erkennen von äquivalenten Ausdrücken (acht Items, vgl. Ball et al. 2003), zum Erkennen von äquivalenten Gleichungen (acht Items, vgl. Steinberg et al. 1991) und zum Bestimmen der Anzahl der Lösungen einer Gleichung (vier Items, vgl. Huntley et al. 2007).

Die Skalenreliabilität (Cronbach’s Alpha, vgl. Taber 2018) für den Pre-, den Post- und den Follow-up-Test unterscheidet sich hinsichtlich der Skalen. Für das prozedurale Wissen ist sie akzeptabel (0,71 < α < 0,76), für die Strategienutzung hoch (0,85 < α < 0,89), für das Strategiewissen akzeptabel bis hoch (0,79 < α < 0,84) und für das konzeptuelle Wissen niedrig (0,52 < α < 0,62). Die Leistungstests wurden von drei geschulten Codierern ausgewertet. 20 % der Stichprobe wurden von zwei Personen codiert, wobei die Intercoder-Übereinstimmung mit κ = 0,92 (Cohen 1960) sehr gut war.

### Statistische Auswertung

Die mit dem Kategoriensystems für die Aufgabenprompts (vgl. Tab. 1) ermittelte Anzahl der angebotenen Aufgaben pro Kategorie und Klasse sind die Prädiktoren auf Klassenebene, für die im Folgenden beschriebenen Regressionsmodelle. Über alle drei Messzeitpunkte (Pre‑, Post- und Follow-up-Test) fehlen zwischen 5 und 9 % der Werte. Der Grund dafür ist, dass Schülerinnen und Schüler bei einem oder mehreren Leistungstests abwesend waren. Somit handelt es sich um zufällig fehlende Werte (*missing at random* [MAR]), die durch multiple Imputationen abgeschätzt wurden (Graham 2008). Ein etabliertes Verfahren zur Imputation fehlender Werte ist MICE (*Multivariate Imputation by Chained Equations,* van Buuren 2012). Die fehlenden Werte in den Leistungsdaten der vorliegenden Studie wurden in sieben Imputationen zu je sieben Iterationsschritten mit dem gleichnamigen R‑Package MICE (van Buuren 2012) geschätzt. Basierend auf diesen imputierten Datensätzen wurde anhand von linearen, hierarchischen Regressionsanalysen geschätzt, wie gut die Anzahl der angebotenen Aufgaben einer Kategorie den Leistungszuwachs der Lernenden voraussagt.

**Abb. 3:**
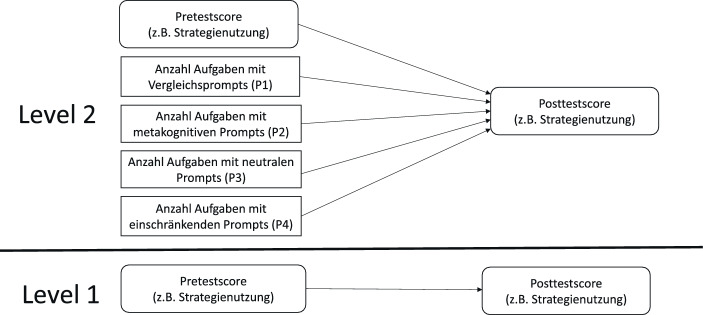
Regression des Posttestscores auf die Anzahl der Aufgaben je Promptkategorie unter Kontrolle der Pretestleistung (vgl. Regressionsgleichungen); dieses Modell wurde für den Leistungszuwachs zwischen Pre- und Posttest wie auch zwischen Pre- und Follow-up-Test für alle vier Skalen (Strategienutzung, Strategiewissen, prozedurales und konzeptuelles Wissen) gerechnet

Aufgrund der Korrelation innerhalb der einzelnen Klassen (die *ICC* liegt je nach Leistungsskala zwischen 0,16 und 0,42, was einer moderaten bis hohen Korrelation entspricht), muss die hierarchische Struktur der Daten berücksichtigt werden. Um die Auswirkungen von Kontexteffekten einzukalkulieren, werden die auf der Individualebene gemessenen Leistungsvariablen in je eine latente Variable auf beiden Levels zerlegt (Lüdtke et al. 2008). Abb. 3 steht stellvertretend für alle im Rahmen dieser Arbeit geschätzten Modelle, die alle dieselbe Struktur aufweisen.

#### Level 1 (within)

$$Po_{ij}=\beta _{0j}+\beta _{\textit{within}}R_{Pr,ij}+\varepsilon _{ij}$$

Neben dem Level-2-Anteil der Kovariate (*U*_*Pr*,*j*_) besteht die Regressionsgleichung für die Klassenebene auch aus den vier Prädiktoren (Anzahl der Aufgaben pro Kategorie):

#### Level 2 (between)

$$\beta _{0j}=\gamma _{00}+\beta _{\textit{between}}U_{Pr,j}+\gamma _{01}\cdot P1+\gamma _{02}\cdot P2+\gamma _{03}\cdot P3+\gamma _{04}\cdot P4+\delta _{j}$$

Die griechischen Buchstaben β und γ stehen für Regressionskoeffizienten bzw. die Intercepts auf beiden Levels. Berichtet werden sowohl standardisierte wie auch nicht standardisierte Regressionsgewichte. Die Prädiktoren auf Klassenlevel sind *P1* (Anzahl der Aufgaben mit Vergleichsprompts), *P*2 (Anzahl Aufgaben mit metakognitiven Prompts), *P3* (Anzahl der Aufgaben mit neutralen Prompts) sowie *P4* (Anzahl der Aufgaben mit einschränkenden Prompts). Geschätzt wurden die Modelle mit dem R‑Package lavaan (Rosseel 2012) unter Verwendung des robusten Schätzers MLR. Dieser wurde gewählt, weil die Plots der Residuen teilweise eine leichte Tendenz zur Heteroskedastizität zeigten.

Das Regressionsmodell enthält fünf Prädiktoren auf Klassenebene und die Stichprobe besteht aus 39 Klassen. Aufgrund dieses Verhältnisses können Überanpassungsprobleme resultieren, was sich in zu großen *R*^2^-Werten niederschlagen kann. Aus diesem Grund werden auch die korrigierten Werte ($R_{korr}^{2}$) berichtet, zudem die Regressionskoeffizienten, die resultieren, wenn neben der Kontrollvariablen nur ein Prädiktor berücksichtigt wird (vgl. Onlineanhang B). In diesen Modellen ist die Gefahr von Überanpassung viel kleiner. Darüber hinaus werden auch die Toleranzwerte für die Prädiktoren bestimmt, um allfällige Kollinearitäten festzustellen (Wolf und Best 2010). Dazu wurde ein flaches Modell (nur Level-2-Variablen) geschätzt, wobei die Leistungsscores manifest auf Klassenebene aggregiert wurden.

Um die Effektstärken einzelner Prädiktoren zu beurteilen, wurde für ausgewählte signifikante Prädiktoren Cohens *f*^2^ (Cohen 1988) berechnet. Schließlich wurde auch die aufgeklärte Varianz (*R*^2^) berechnet, wofür zwei weitere Modelle verwendet wurden. Beim einen wurden neben der Kontrollvariablen (Leistung im Pretest) keine Prädiktoren aufgenommen; im anderen Modell wurde als einziger Prädiktor die Anzahl aller in einer Klasse angebotenen Aufgaben (also die Summe von *P1* bis *P4*) berücksichtigt.

## Resultate

Bevor die Ergebnisse der Regressionsanalysen berichtet werden, folgt nun ein Überblick über die Leistungsdaten der Schülerinnen und Schüler sowie über die in den Klassen angebotenen Aufgaben.

### Deskriptive Analysen

**Abb. 4:**
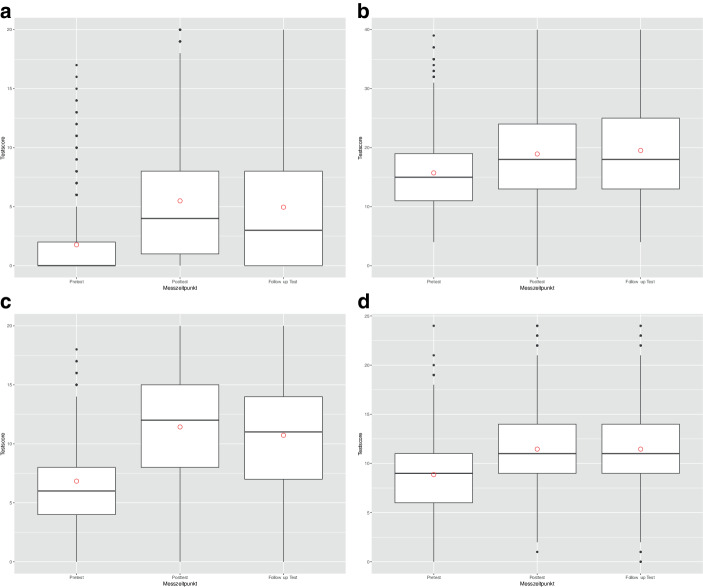
Verteilung der Testscores zu drei Messzeitpunkten (die Kringel stehen für die Mittelwerte). **a** Strategienutzung (Punktemaximum: 20); **b** Strategiewissen (Punktemaximum: 40); **c** Prozedurales Wissen (Punktemaximum: 20); **d** Konzeptuelles Wissen (Punktemaximum: 24)

Auf allen vier Leistungsskalen wurde ein Leistungszuwachs zwischen dem Pre- und dem Posttest sowie zwischen dem Pre- und dem Follow-up-Test gemessen (vgl. Abb. 4 und Onlineanhang C).

Bei der Strategienutzung zeigen sich im Gegensatz zu den anderen Wissensbereichen Bodeneffekte. Verhältnismäßig kleine Leistungszuwächse wurden beim Strategiewissen (vgl. Abb. 4b) und im Bereich des konzeptuellen Wissens (Abb. 4d) erzielt. Was das prozedurale Wissen betrifft (Abb. 4c), kann hingegen ein starker Zuwachs zwischen dem Pre- und dem Posttest beobachtet werden. Eine Tabelle mit den Effektstärken findet sich im Onlineanhang D.

Um zu beurteilen, ob die festgestellten Leistungszuwächse auf das Potenzial der angebotenen Aufgaben für kognitive Aktivierung zurückzuführen sind, wurde die Anzahl der Aufgaben pro Kategorie und Klasse erfasst. Zwischen den Klassen zeigten sich bezüglich der Anzahl der Aufgaben aller Kategorien erhebliche Unterschiede (vgl. Tab. 2).

**Tab. 2:** Mittelwerte (Standardabweichungen) sowie Minima und Maxima der Anzahl in einer Klasse angebotener Aufgaben je Hauptkategorie der Aufgabenprompts

Variable	Mittelwert	(SD)	Minimum	Maximum
Einschränkende Prompts	20,56	(19,02)	0	99
Neutrale Prompts	27,14	(19,58)	0	80
Metakognitive Prompts	26,44	(31,68)	0	142
Vergleichsprompts	2,98	(3,48)	0	12

Im Mittel wurden während der Unterrichtseinheit 77 Aufgaben angeboten (*SD* = 43,5), wobei die Spannweite 201 betrug (*min* = 14/*max* = 215).

**Abb. 5:**
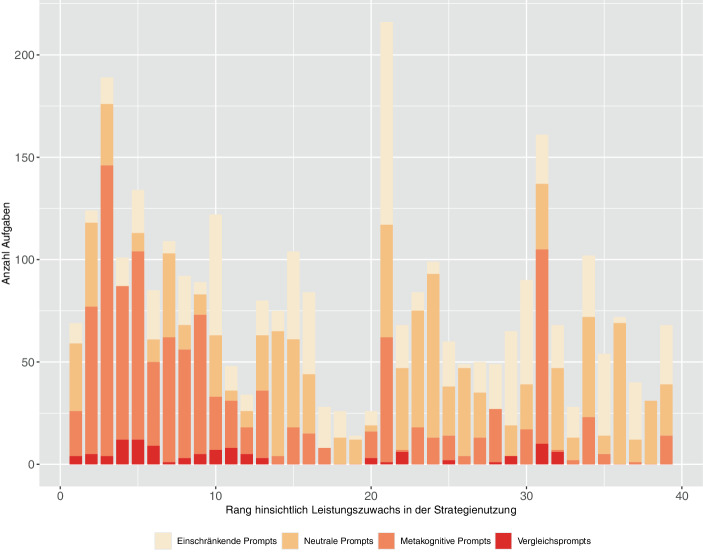
Häufigkeit der in den 39 Projektklassen angebotenen Aufgaben, unterteilt in die vier Promptkategorien und in absteigender Reihenfolge geordnet nach dem Leistungszuwachs in der Strategienutzung (Rang 1 bedeutet, dass der Zuwachs am größten ist)

Die Gesamtanzahl der in den Klassen angebotenen Aufgaben variiert stark. Das gilt auch für die Anzahl der Aufgaben pro Kategorie. In Abb. 5 ist dies exemplarisch für den Leistungszuwachs in der Strategienutzung ersichtlich. Die Säulen sind nach dem Klassenmittelwert des Leistungszuwachses in absteigender Reihenfolge angeordnet. Die Säule ganz links zeigt das Aufgabenangebot der Klasse mit dem größten Leistungszuwachs in der Strategienutzung. Diese Anordnung macht deutlich, dass in Klassen mit großen Leistungszuwächsen in der Strategienutzung tendenziell mehr Aufgaben mit Vergleichsprompts und metakognitiven Prompts angeboten wurden.

### Abhängigkeit des Leistungszuwachses von den Promptkategorien

Im Folgenden werden die Resultate der multiplen linearen Zweiebenenregression dargestellt (vgl. Tab. 3). Die Analyse der Scatterplots wie auch der Residualplots lassen abgesehen von einer leichten Tendenz zur Heteroskedastizität keine Verletzungen der Voraussetzungen für lineare Regressionen erkennen. Aufgrund dieses Befundes wurde ein robuster Schätzer für die Berechnungen verwendet. In den Modellen ohne Kontrolle der Teilnahme an den Weiterbildungen (vgl. Resultate in Tab. 3) liegen keine problematischen Kollinearitäten vor, die Toleranzwerte (Wolf und Best 2010) sind alle größer als 0,5 (bzw. *VIF* < 2). Unter Kontrolle der Weiterbildungen liegt der tiefste Toleranzwert für *P1* (Anzahl der Aufgaben mit Vergleichsprompts) bei 0,44 (*VIF* = 2,26), was nicht auf problematische Kollinearitäten hindeutet. Zwischen den Variablen *P1* und *EG_Vergl* liegt ein signifikanter, moderater Zusammenhang vor (*r* = 0,4, *p* < 0,05), während für *P1* und *EG_Vergl & AT* kein signifikanter Zusammenhang vorhanden ist.

**Tab. 3:** Level-2-Regressionskoeffizienten (Standardfehler) zur Vorhersage von Post- und Follow-up-Testscores unter Kontrolle der Pretestscores

	Regressionskoeffizienten (zwischen Pre- und Posttestscore)			Regressionskoeffizienten (zwischen Pre- und Follow-up-Testscore)		
Prädiktor	Unstandardisiert (Standardfehler)		Standardisiert	Unstandardisiert (Standardfehler)		Standardisiert
*Strategienutzung*						
γ_00_ (Intercept)	2,02	(0,60)***	0,61	2,81	(0,68)***	0,91
Β_between_ (Pretestscore)	1,31	(0,17)***	0,69	1,34	(0,23)***	0,75
γ_01_ (Vergleichsprompts)	0,23	(0,09)**	0,24	0,16	(0,09)*	0,18
γ_02_ (metakognitive Prompts)	0,03	(0,01)***	0,34	0,02	(0,01)*	0,17
γ_03_ (neutrale Prompts)	0,00	(0,01)	0,01	−0,02	(0,01)	−0,14
γ_04_ (einschränkende Prompts)	−0,02	(0,01)*	−0,13	−0,03	(0,01)**	−0,17
*Strategiewissen*						
γ_00_ (Intercept)	3,77	(1,39)**	0,87	4,99	(1,32)***	1,13
Β_between_ (Pretestscore)	1,02	(0,07)***	0,87	1,04	(0,06)***	0,87
γ_01_ (Vergleichsprompts)	0,01	(0,11)	0,01	0,02	(0,10)	0,02
γ_02_ (metakognitive Prompts)	0,03	(0,01)**	0,19	0,01	(0,01)	0,06
γ_03_ (neutrale Prompts)	−0,03	(0,01)*	−0,15	−0,04	(0,02)**	−0,17
γ_04_ (einschränkende Prompts)	−0,03	(0,01)*	−0,13	−0,05	(0,01)***	−0,19
*Prozedurales Wissen*						
γ_00_ (Intercept)	3,43	(1,80)†	1,73	4,58	(2,41) †	2,12
Β_between_ (Pretestscore)	1,00	(0,21)***	0,82	0,95	(0,26)***	0,71
γ_01_ (Vergleichsprompts)	0,21	(0,09)*	0,37	0,07	(0,12)	0,11
γ_02_ (metakognitive Prompts)	0,01	(0,01)†	0,16	0,01	(0,01)	0,08
γ_03_ (neutrale Prompts)	0,01	(0,01)	0,12	−0,02	(0,02)	−0,16
γ_04_ (einschränkende Prompts)	−0,00	(0,01)	−0,04	−0,01	(0,02)	−0,11
*Konzeptuelles Wissen*						
γ_00_ (Intercept)	2,23	(1,40)	1,47	1,55	(1,57)	0,88
Β_between_ (Pretestscore)	0,96	(0,15)***	0,92	1,11	(0,16)***	0,91
γ_01_ (Vergleichsprompts)	0,02	(0,05)	0,05	0,05	(0,07)	0,11
γ_02_ (metakognitive Prompts)	0,01	(0,00)**	0,25	0,00	(0,01)	0,08
γ_03_ (neutrale Prompts)	0,01	(0,01)	0,13	−0,00	(0,01)	−0,03
γ_04_ (einschränkende Prompts)	0,00	(0,01)	0,01	−0,00	(0,01)	−0,04

Hinweis: Die *p*-Werte für die Prädiktoren wurden den gerichteten Hypothesen entsprechend mit einem einseitigen Hypothesentest ermittelt

*p < 0,05, **p < 0,01, ***p < 0,001, † *p* < 0,1

#### Anzahl der Aufgaben mit Vergleichsprompts (Hypothese 1)

Signifikante Abhängigkeiten des Leistungszuwachses von der Anzahl der im Unterricht angebotenen Aufgaben mit Vergleichsprompts wurden in der Strategienutzung (γ_01_ = 0,24, *t* = 2,41, *p* = 0,008) und im Bereich des prozeduralen Wissens (γ_01_ = 0,37, *t* = 2,19, *p* = 0,015) festgestellt (vgl. Tab. 3). Wenn die Teilnahme an einer der beiden Weiterbildungen statistisch kontrolliert wird, liegen diese Abhängigkeiten jedoch nicht vor (vgl. Tab. 4).

**Tab. 4:** Level-2-Regressionskoeffizienten (Standardfehler) zur Vorhersage von Post- und Follow-up-Testscores unter Kontrolle der Pretestscores und der Teilnahme an einer Weiterbildung

	Regressionskoeffizienten (zwischen Pre- und Posttestscore)			Regressionskoeffizienten (zwischen Pre- und Follow-up-Testscore)		
Prädiktor	Unstandardisiert (Standardfehler)		Standardisiert	Unstandardisiert (Standardfehler)		Standardisiert
*Strategienutzung*						
γ_00_ (Intercept)	1,55	(0,47)**	0,47	2,29	(0,53)***	0,74
Β_between_ (Pretestscore)	1,19	(0,13)***	0,62	1,29	(0,20)***	0,73
γ_01_ (Vergleichsprompts)	0,09	(0,10)	0,10	0,08	(0,11)	0,09
γ_02_ (metakognitive Prompts)	0,03	(0,01)***	0,34	0,02	(0,01)*	0,16
γ_03_ (neutrale Prompts)	0,00	(0,01)	0,00	−0,02	(0,01)	−0,11
γ_04_ (einschränkende Prompts)	−0,02	(0,01)*	−0,11	−0,02	(0,01)**	−0,15
γ_05_ (EG_Vergl)	1,80	(0,77)*	0,26	1,30	(0,83)†	0,20
γ_06_ (EG_Vergl & AT)	1,38	(0,75)*	0,20	0,71	(0,80)	0,11
*Strategiewissen*						
γ_00_ (Intercept)	3,80	(1,32)**	0,89	4,42	(1,47)**	0,99
Β_between_ (Pretestscore)	1,00	(0,07)***	0,84	1,04	(0,08)***	0,86
γ_01_ (Vergleichsprompts)	−0,03	(0,12)	−0,03	0,01	(0,12)	0,01
γ_02_ (metakognitive Prompts)	0,02	(0,01)**	0,17	0,01	(0,01)	0,06
γ_03_ (neutrale Prompts)	−0,03	(0,01)*	−0,13	−0,03	(0,02)*	−0,14
γ_04_ (einschränkende Prompts)	−0,03	(0,01)**	−0,14	−0,04	(0,01)***	−0,19
γ_05_ (EG_Vergl)	0,75	(0,73)	0,08	0,47	(0,82)	0,05
γ_06_ (EG_Vergl & AT)	0,70	(0,81)	0,08	0,29	(1,00)	0,03
*Prozedurales Wissen*						
γ_00_ (Intercept)	4,19	(1,72)*	2,12	4,04	(2,36)†	1,86
Β_between_ (Pretestscore)	0,84	(0,20)***	0,68	0,99	(0,28)***	0,75
γ_01_ (Vergleichsprompts)	0,10	(0,10)	0,18	0,08	(0,13)	0,12
γ_02_ (metakognitive Prompts)	0,01	(0,01)*	0,15	0,01	(0,01)	0,07
γ_03_ (neutrale Prompts)	0,01	(0,01)	0,09	−0,01	(0,02)	−0,13
γ_04_ (einschränkende Prompts)	−0,01	(0,01)	−0,06	−0,01	(0,02)	−0,08
γ_05_ (EG_Vergl)	1,34	(0,65)*	0,32	0,28	(0,89)	0,06
γ_06_ (EG_Vergl & AT)	1,09	(0,72)†	0,26	−0,04	(0,91)	−0,01
*Konzeptuelles Wissen*						
γ_00_ (Intercept)	2,37	(1,57)	1,65	−0,21	(1,85)	−0,12
Β_between_ (Pretestscore)	0,94	(0,18)***	0,94	1,33	(0,20)***	1,07
γ_01_ (Vergleichsprompts)	0,05	(0,07)	0,12	0,16	(0,10)†	0,31
γ_02_ (metakognitive Prompts)	0,01	(0,00)**	0,22	0,01	(0,01)	0,09
γ_03_ (neutrale Prompts)	0,01	(0,01)	0,16	0,00	(0,01)	0,02
γ_04_ (einschränkende Prompts)	−0,00	(0,01)	−0,03	−0,00	(0,01)	−0,04
γ_05_ (EG_Vergl)	−0,28	(0,63)	−0,09	−1,40	(0,73)*	−0,37
γ_06_ (EG_Vergl & AT)	0,41	(0,48)	0,14	−0,80	(0,69)	−0,21

Hinweis: Die *p*-Werte für die Prädiktoren wurden den gerichteten Hypothesen entsprechend mit einem einseitigen Hypothesentest ermittelt. Das gilt auch für die Kontrollvariablen der Versuchsgruppen. (Die Wirksamkeit der Weiterbildungen wurden in der Studie von Rüede et al.(2023) untersucht)

*p < 0,05, **p < 0,01, ***p < 0,001, † *p* < 0,1

#### Anzahl der Aufgaben mit metakognitiven Prompts (Hypothese 2)

Der Leistungszuwachs hängt außer im Bereich des prozeduralen Wissens in allen Wissensbereichen von der Anzahl der angebotenen Aufgaben mit metakognitiven Prompts ab (vgl. Tab. 3). Für diese drei Variablen kann somit die Hypothese 2 gestützt werden. Die Effektstärken der drei signifikanten Prädiktoren liegen zwischen *f*^*2*^ = 0,27 (Strategienutzung) und *f*^*2*^ = 0,33 (Strategiewissen). Es handelt sich somit um mittelstarke bis starke Effekte. Diese signifikanten Abhängigkeiten bleiben auch bestehen, wenn die Teilnahme an den Weiterbildungen kontrolliert wird (vgl. Tab. 4).

#### Anzahl der Aufgaben mit neutralen Prompts (Hypothese 3)

Aufgrund des nicht signifikanten Regressionsgewichtes (vgl. Tab. 3) kann Hypothese 3 für die Abhängigkeit des Leistungszuwachses im Bereich des prozeduralen Wissens von der Anzahl der Aufgaben mit neutralen Prompts nicht gestützt werden. Als exploratives Ergebnis, das über die formulierten Hypothesen hinausgeht, kann festgestellt werden, dass es bezüglich des Leistungszuwachses im Strategiewissen zwischen Pre- und Posttest signifikante negative Abhängigkeiten gibt (vgl. Tab. 3).

#### Anzahl der Aufgaben mit einschränkenden Prompts (Hypothese 4)

Aus den Regressionsanalysen (vgl. Tab. 3) resultierten hypothesenkonform signifikante negative Regressionskoeffizienten für die Anzahl der Aufgaben mit einschränkenden Prompts auf den Leistungszuwachs zwischen dem Pre- und dem Posttest in den Bereichen Strategienutzung (γ_04_ = −0,13, *t* = −2,14, *p* = 0,017) und Strategiewissen (γ_04_ = −0,13, *t* = −2,31, *p* = 0,011). Bei der Strategienutzung ist der Effekt schwach (*f*^*2*^ = 0,07), beim Strategiewissen liegt ein mittlerer Effekt (*f*^*2*^ = 0,23) vor. Das Resultat bleibt unter Kontrolle der Teilnahme an den Weiterbildungen bestehen. Für die Strategienutzung konnte es im sequenziellen Modell (vgl. Onlineanhang B) mit nur einem Prädiktor jedoch nicht bestätigt werden, somit muss es vorsichtig interpretiert werden. Was den Bereich des prozeduralen Wissens betrifft, konnten hingegen keine signifikanten förderlichen Auswirkungen der Anzahl der Aufgaben mit einschränkenden Prompts festgestellt werden.

#### Varianzaufklärung

Dass sich die im Rahmen dieser Arbeit entwickelte Kategorisierung der Aufgabenprompts und die Regressionsmodelle zur Beantwortung der Fragestellungen bewähren, zeigt sich auch im Bestimmtheitsmaß (*R*^2^). Um die Modellgüte der geschätzten Modelle (vgl. Abb. 3) zu ermitteln, wurde ein weiteres Modell ohne Prädiktoren geschätzt (Tab. 5, Spalte 1). Aufgrund des Verhältnisses zwischen der Anzahl der Prädiktoren und der Anzahl der Fälle (39) auf Klassenebene kann ein direkter Vergleich der Bestimmtheitsmaße zwischen Modellen mit einer unterschiedlichen Anzahl von Prädiktoren problematisch sein (Wolf und Best 2010). Aus diesem Grund wird auch das korrigierte Bestimmtheitsmaß $R_{korr}^{2}$ in Klammern angegeben (vgl. Tab. 5).

**Tab. 5:** Bestimmtheitsmaß R^**2**^ ($\boldsymbol{R}_{\boldsymbol{korr}}^{\mathbf{2}}$) für die Regressionsmodelle für den Leistungszuwachs zwischen Pre- und Posttest

Prädiktoren		Anzahl Aufgaben der Kategorien 1–4	Anzahl Aufgaben der Kategorien 1–4
Kontrollvariablen	Pretestleistung	Pretestleistung	Pretestleistung, Versuchsgruppen
Anzahl UV	1	5	7
Strategienutzung	0,49 (0,47)	0,77 (0,73)	0,81 (0,77)
Strategiewissen	0,86 (0,86)	0,93 (0,92)	0,93 (0,91)
Prozedurales Wissen	0,50 (0,48)	0,70 (0,65)	0,73 (0,67)
Konzeptuelles Wissen	0,78 (0,77)	0,87 (0,85)	0,88 (0,85)

Bei allen vier Regressionsmodellen wird ein sehr großer Varianzanteil durch die Pretestleistung erklärt, was die erste Spalte von Tab. 5 zeigt. Besonders auffällig ist dies beim Strategiewissen und im Bereich des konzeptuellen Wissens. Durch die Modelle ohne Kontrolle der Weiterbildung wird insbesondere im Falle der Strategienutzung und des prozeduralen Wissens ein zusätzlicher Varianzanteil aufgeklärt (Tab. 5, Spalte 2 im Vergleich zu Spalte 1). Wird nun zusätzlich die Teilnahme an der Weiterbildung kontrolliert, ist der Gewinn an Varianzaufklärung nur gering (Tab. 5, letzte Spalte).

## Diskussion

Im Rahmen dieser Arbeit wurde das Potenzial zur kognitiven Aktivierung unterschiedlicher Prompts von Aufgaben zum regelbasierten Lösen quadratischer Gleichungen untersucht. Im Zentrum stand die Frage, welche Aufgabenprompts den Zuwachs in Strategieflexibilität (Strategienutzung und Strategiewissen) sowie in den Bereichen prozedurales und konzeptuelles Wissen erklären.

### Zusammenfassung und Einordnung der Ergebnisse

Aus der Literatur (z. B. Cohors-Fresenborg et al. 2010; Hattie 2023; Hiebert und Grouws 2007; Kunter et al. 2011; Lipowsky et al. 2009; Stein und Lane 1996; Stillman und Mevarech 2010) ist bekannt, dass ein (meta‑)kognitiv aktivierender Unterricht sich positv auf den Leistungszuwachs auswirken kann, und dass Aufgaben bei der Aktivierung der Lernenden eine zentrale Rolle spielen. Mit der vorliegenden Untersuchung wurde gezeigt, dass auch beim regelbasierten Lösen von quadratischen Gleichungen der Leistungszuwachs von der Qualität und Häufigkeit des Aufgabenangebots abhängt. So wurde hypothesenkonform gezeigt, dass a) der Leistungszuwachs in der Strategienutzung und im Bereich des prozeduralen Wissens positiv von den im Unterricht angebotenen Vergleichsaufgaben abhängt, b) dass der Leistungszuwachs in der Strategienutzung, im Strategiewissen und im Bereich des konzeptuellen Wissens positiv von den Aufgaben mit metakognitiven Prompts und c) dass der Leistungszuwachs in der Strategieflexibilität negativ von den Aufgaben mit einschränkenden Prompts abhängt.

Vorangehende Studien (Durkin et al. 2023; Rittle-Johnson und Star 2007, 2009) legten nahe, dass der Leistungszuwachs beim regelbasierten Lösen von Gleichungen unterstützt wird, wenn Aufgaben bearbeitet werden, die zum Vergleichen vorgegebener Lösungswege auffordern; ein empirischer Nachweis im realen Mathematikunterricht fehlte aber bislang. Mit der vorliegenden Studie wurde über das Vergleichen hinausgehend die Abhängigkeit des Leistungszuwachses von der Qualität und der Quantität der angebotenen Aufgaben beim Lösen von Gleichungen erstmals systematisch untersucht. Besonders hervorzuheben ist der empirische Befund, dass der Leistungszuwachs beim regelbasierten Lösen von quadratischen Gleichungen auch von der Anzahl der Aufgaben mit metakognitiven Prompts abhängt.

Vor diesem Hintergrund rückt die Rolle metakognitiver Prompts in den Fokus, insbesondere in Bezug auf deren Potenzial, Vergleichsprozesse beim Lösen von Gleichungen anzuregen. Ähnlich wie Vergleichsprompts sollen metakognitive Prompts beim Generieren eigener Lösungswege die Aufmerksamkeit auf Planungs- und Evaluationsprozesse lenken. Dabei werden vermutlich antizipierte Lösungswege miteinander verglichen. Das hieße, dass im Unterricht Vergleichsprozesse nicht nur durch Vergleichsprompts, sondern auch durch metakognitive Prompts initiiert werden könnten. Vermutlich kann so mittels metakognitiver Prompts die Integration des Vergleichens in den realen Mathematikunterricht unterstützt werden.

Bedeutsam für den Mathematikunterricht ist weiter, dass sich Aufgaben mit einschränkenden Prompts negativ auf die Entwicklung der Strategieflexibilität auswirken. Zudem zeigen die Resultate über die formulierten Hypothesen hinausgehend, dass die Anzahl der angebotenen Aufgaben mit neutralen Prompts negativ mit der Entwicklung des Strategiewissens zusammenhängt.

Die Abhängigkeit des Zuwachses in der Strategieflexibilität von der Anzahl der Aufgaben mit Vergleichsprompts ist nicht mehr feststellbar, wenn die Teilnahme an einer der beiden Weiterbildungen kontrolliert wird. Weil die bivariaten Korrelationen zwischen den Prädiktoren alle deutlich unter $r=.7$ (vgl. Tabachnick und Fidell 2013, S. 90) liegen, kann dieser Befund höchstens teilweise auf Multikollinearitäten zurückgeführt werden. Der Grund für den Wegfall der Abhängigkeit dürfte darin liegen, dass das spezifische Format der Vergleichsaufgaben erst durch die Weiterbildung in den realen Mathematikunterricht gebracht wurde. Denn in der gesamten Kontrollgruppe kam nur eine einzige solche Aufgabe vor (Rüede et al. 2023). Daher erklärt die Teilnahme an der Weiterbildung das Vorkommen von Aufgaben mit Vergleichsprompts.

Nicht gestützt wird Hypothese 1 für das Strategiewissen und das konzeptuelle Wissen. Möglicherweise reicht die Verwendung von Vergleichsprompts im Unterricht nicht aus, um Lernprozesse in diesen beiden Bereichen anzuregen. Ob Lernende auch diesbezüglich Fortschritte erzielen, kann auch von der weiteren Unterrichtsgestaltung abhängen. Beispielsweise legen die Resultate von Mok et al. (2022) nahe, dass die Wirkung von Vergleichsprompts erhöht wird, wenn die Lösungswege in produktiv geführten Klassengesprächen (*accountable talk*, vgl. Michaels et al. 2013) verglichen werden. Besonders die in der Klasse generierten Schülerbegründungen waren für die Entwicklung des konzeptuellen Wissens bedeutsam.

Die Bestätigung von Hypothese 2 für alle Wissensformen mit Ausnahme des prozeduralen Wissens deutet darauf hin, dass metakognitive Anregungen den Leistungszuwachs fördern können. Vor diesem Hintergrund erscheinen Konzeptualisierungen plausibel, die Anregungen zur Metakognition als Bestandteil des kognitiven Aktivierungspotenzials von Aufgaben verstehen (Widmer et al. 2024).

Zwischen dem Leistungszuwachs im Bereich des prozeduralen Wissens und der Anzahl der Aufgaben mit neutralen Prompts sowie der Anzahl der Aufgaben mit einschränkenden Prompts konnte keine signifikante Abhängigkeit nachgewiesen werden (Hypothesen 3 und 4), obwohl sie implizit oder explizit zum Lösen auffordern und so auf die Entwicklung des prozeduralen Wissens abzielen. Möglicherweise ist dieser Befund auf eine Sättigung zurückzuführen (vgl. Abschn. 5.2 Limitationen).

Bislang wurde der positive Einfluss des Vergleichens auf den Leistungszuwachs beim Gleichungslösen für lineare Gleichungen (Durkin et al. 2023; Rittle-Johnson und Star 2007, 2009) und quadratische Gleichungen (Rüede et al. 2023) gezeigt. Es wird vermutet, dass bei anderen Gleichungstypen vergleichbare Zusammenhänge zwischen dem Aufgabenangebot und dem Leistungszuwachs bestehen. Denn andere Gleichungstypen umfassen auch Gleichungen, die sich auf unterschiedlich effizienten Wegen lösen lassen (für Bruchtermgleichungen vgl. z. B. Rüede 2015).

### Limitationen

Eine Limitation dieser Studie betrifft die Stichprobe. Sie stammt aus einer Interventionsstudie, an der die Lehrkräfte freiwillig teilnahmen. Somit handelt es sich nicht um eine zufällig gezogene Stichprobe aus der Grundgesamtheit. Diese Limitation musste in Kauf genommen werden, weil erst durch die experimentelle Intervention untersucht werden konnte, wie sich das Vergleichen von Lösungswegen auf den Leistungszuwachs auswirkt. Denn ohne die Weiterbildung nutzen die Lehrkräfte kaum Aufgaben mit Vergleichsprompts, wie die Analyse der verwendeten Aufgaben in der Kontrollgrupe gezeigt hat (Rüede et al. 2023).

Möglicherweise unterscheiden sich die im Unterricht diskutierten Gleichungen hinsichlich des Anspruchniveaus zwischen den Weiterbildungsgruppen, sodass sich dies auf das Potenzial zur kognitiven Aktivierung auswirkte. Um in dieser Hinsicht einen vergleichbaren Orientierungsrahmen zu bieten, wurden alle Lehrkräfte über die zu erreichenden Minimalziele informiert. Diese Ziele wurden anhand von sechs Beispielgleichungen sowie Hinweisen auf gängige Lehrmittel konkretisiert. Sollte trotz dieses Orientierungsrahmens das Anspruchsniveau der angebotenen Gleichungen zwischen den Versuchsgruppen bzw. den Klassen nicht vergleichbar gewesen sein, ist ein bedeutsamer Einfluss auf die Resultate in diesem Kontext dennoch nicht zu vermuten. Denn ob das Nachdenken über Lösungswege angeregt wird, hängt wesentlich von den Aufgabenprompts ab (Sjuts 2022).

Auch zu berücksichtigen ist, dass Aufgaben, die eine vertiefte Auseinandersetzung anregen, mehr Bearbeitungszeit benötigen als solche mit neutralen Prompts. Eine weitere Limitation der vorliegenden Studie ist somit, dass die tatsächlich aufgewendete Zeit *(time-on-task)* pro Aufgabe nicht erfasst wurde. Vor dem Hintergrund, dass allen Klassen für die Bearbeitung des Themas ein identischer Zeitraum von sechzehn Unterrichtsstunden zur Verfügung stand, erscheint es vertretbar, diese Limitation in Kauf zu nehmen.

Wie bereits im Methodenteil (vgl. Abschn. 3.5) erörtert, ist das Verhältnis zwischen der Anzahl der Fälle und der Anzahl der Prädiktoren auf dem Klassenlevel möglicherweise ungünstig. Die vergleichsweise geringe Anzahl der Klassen (*N* = 39) im Verhältnis zu den vier Prädiktoren und einer Kovariaten auf Klassenebene kann dazu führen, dass die Effekte überschätzt werden. Aus diesem Grund wurden auch Modelle mit nur je einem Prädiktor gerechnet (vgl. Onlineanhang B). Auf diese Weise wurden die Resultate mit einer Ausnahme bestätigt: Dass sich die Anzahl der Aufgaben mit einschränkenden Prompts negativ auf die Strategienutzung auswirkt, wurde in der sequenziellen Modellierung nicht gestützt. Daher sollte dieses Ergebnis vorsichtig interpretiert werden. Das gilt auch für die in Tab. 4 berichteten Resultate. Die Kontrolle der Teilnahmen an den Weiterbilungen erschwert die Interpretation der Resultate aufgrund möglicher Überanpassungseffekte.

Auch kann infrage gestellt werden, ob eine lineare Modellierung sich eignet, um die Hypothesen zu testen. Durch Hinzunahme von quadratischen Termen in das Regressionsmodell könnten allfällige Sättigungseffekte durch sehr viele Aufgaben ein und derselben Promptkategorie aufgedeckt werden. Auf Basis der im Rahmen dieser Untersuchung verwendeten Daten ist es aber nur eingeschränkt möglich, belastbare Aussagen zur Sättigung zu machen, weil sich die beschriebene Problematik von Überanpassungseffekten unter Einbezug weiterer, quadratischer Terme in das Regressionsmodell verstärken kann. Trotz der eingeschränkten Aussagekraft wurden auch Modelle unter Berücksichtigung quadratischer Terme geschätzt. Diese quadratische Modellierung führte vereinzelt zu signifikanten Abhängigkeiten, wobei sowohl der lineare als auch der quadratische Term signifikant waren. Dies deutet auf eine Sättigung hin. Bemerkenswert ist, dass der Leistungszuwachs im Bereichs des prozeduralen Wissens signifikant von der Anzahl Aufgaben mit neutralen Prompts abängt, wenn der quadratische Term aufgenommen wird (negatives quadratisches, positives lineares Regressionsgewicht). Diese Abhängigkeit wurde mit der linearen Modellierung (vgl. Abschn. 4.2) nicht festgestellt, was darauf hindeutet, dass sich Aufgaben mit neutralen Prompts zur Förderung des prozeduralen Wissen eignen, sich aber eine Sättigung einstellt.

Eine weitere Limitation dieses Beitrags besteht in der Beschränkung auf das Potenzial zur kognitiven Aktivierung. Weder die effektive Nutzung der Lernzeit noch andere Aspekte der Aufgabenimplementation wurden gemessen. Durch die randomisierte Zuteilung zu den Versuchsbedingungen ist davon auszugehen, dass entsprechende Unterschiede zwischen den Gruppen ausgemittelt werden.

Die nur knapp akzeptablen Werte der Skalenreliabilität für die Messung des konzeptuellen Wissens (0,52 < α < 0,62) sind vergleichbar mit jenen, die in anderen Studien berichtet werden (z. B. Rittle-Johnson und Star 2007, 2009). Die im Rahmen dieser Arbeit verwendeten Items zur Bewertung des konzeptuellen Wissens wurden eng angelehnt an jene von Rittle-Johnson und Star (2007, 2009) entwickelt, was die vergleichbaren Werte für die Skalenreliabilität erklärt (vgl. dazu auch Rüede et al. 2023). Ein weiterer limitierender Aspekt hinsichtlich der Reliabilität ist die Zeitbeschränkung bei der Erfassung des konzeptuellen Wissens (20 Items in 10 min). Hinzu kommen mögliche Ermüdungseffekte, da entsprechende Items erst gegen Ende des Testes bearbeitet werden mussten. Zudem ist die Operationalisierung des konzeptuellen Wissens auf wenige Teilaspekte beschränkt. Insofern ist auch die Validität dieser Messung möglicherweise eingeschränkt. Insgesamt könnten die vergleichsweise geringen Leistungszuwächse im Bereich des konzeptuellen Wissen (vgl. Abb. 4d) zumindest teilweise auf die Einschränkungen hinsichtlich dieser beiden Gütekriterien zurückzuführen sein.

### Fazit

Die vorliegende Untersuchung leistet einen Beitrag zum besseren Verständnis der Frage, wie der Leistungszuwachs beim regelbasierten Lösen von quadratischen Gleichungen von den Aufgabenprompts abhängt. Es zeigte sich, dass die Anzahl der Aufgaben mit zwei vorgegebenen Lösungswegen und Vergleichsprompts und die Anzahl der Aufgaben mit metakognitiven Prompts den Leistungszuwachs unterstützen, während Aufgaben mit einschränkenden Prompts die Entwicklung der Strategieflexibilität hemmen. Damit im Unterricht zum Lösen von Gleichungen unterschiedliche Lösungswege häufiger initiiert und aufeinander bezogen werden, sind Aufgaben mit zwei vorgegebenen Lösungswegen und Vergleichsprompts sowie solche mit metakognitiven Prompts bei der Aus- und Weiterbildung von Lehrkräften wie auch bei der Weiterentwicklung von Lehrmitteln zu berücksichtigen. Das vorgestellte Kategoriensystem kann als Grundlage für diese Weiterentwicklung dienen.

## Supplementary Information

Onlineanhang

## Footnotes

1 Rittle Johnson und Star (2009) verwenden dafür den Begriff *procedural flexibility.*

2 Rittle Johnson und Star (2009) bezeichnen dies als *flexibility knowledge*, Sievert et al. (2022) als *Kenntnis verschiedener Lösungsstrategien.*

3 Rittle Johnson und Star (2009) bezeichnen dies als *flexibility use*, Heinze et al. (2020) verwenden im Zusammenhang mit Rechenstrategien den Begriff *adaptive Nutzung von Rechenstrategien.*

4 Für eine detaillierte Beschreibung der Kursinhalte vgl. Rüede et al. (2023).
