# Supplementary material for: The Role of Task Prompts in Fostering Strategy Flexibility, Procedural, and Conceptual Knowledge when Solving Quadratic Equations
Source: J Math Didakt. 2025 Aug 18;46(2):11. [Article in German] doi: 10.1007/s13138-025-00262-y (PMC12360982; doi:10.1007/s13138-025-00262-y)
Supplement: Supplementary file 1 — Onlineanhang [file 13138_2025_262_MOESM1_ESM.docx]

# Anhang A: Beispielitems zur Leistungsmessung

Tab. A1: Beispielitems aus den Leistungstests (Rüede et al., 2023) mit Lösungserwartung

| Skala | Beispielitems | Kodierung (erwartete Lösung) |
| --- | --- | --- |
| Prozedurales Wissen  lineare Gleichung (*n* = 2)  quadratische Gleichung (*n* = 8) | Löse die Gleichung nach *x* auf:  $3\left( x+2 \right)+4\left( x+2 \right)=35$  $\left( x-2 \right)^{2}-2=23$ | Korrekte Lösung liegt vor  $x=3$  $x_{1}=-3, x_{2}=7$ |
| Strategienutzung | Die Strategienutzung wurde anhand derselben Gleichungen gemessen wie das prozedurale Wissen. Es wurde beurteilt, ob ein effizienter (aus möglichst wenigen Schritten bestehender) Weg gewählt wurde. | Lösungsschritte:  $7\left( x+2 \right)=35$  $\left( x-2 \right)^{2}=25, x-2=\pm5$ |
| Strategiewissen |  |  |
| Generieren multipler Lösungswege (*n* = 6) | Löse die Gleichung auf zwei möglichst unterschiedliche Arten  $4x\left( x-1 \right)+x\left( x-1 \right)=0$  Falls du zwei Lösungswege hast: Welcher ist deines Erachtens am schnellsten? | Zwei korrekte Lösungswege liegen vor, z. B:  $5x\left( x-1 \right)=0, x_{1}=0, x_{2}=+1$  $5x^{2}-5x=0,$anschl. Lösungsformel  Der Weg ohne Ausmultiplizieren wird als schneller eingestuft. |
| Erkennen multipler Lösungswege (*n* = 16) | Darf der jeweilige Schritt bei der Gleichung $2x\left( x+2 \right)-4\left( x+2 \right)=2x-4$ ausgeführt werden (selbst wenn er nicht viel bringt)?   1. Auf beiden Seiten $2x(x+2)$ addieren 2. Auf der linken Seite ausmultiplizieren 3. Auf der linken Seite $(x+2)$ ausklammern | Bei allen drei Items wird „Ja“ angekreuzt. |
| Beurteilen der Effizienz von Lösungswegen (*n* = 6) | Ein erster Schritt zur Lösung der Gleichung $9\left( x+7 \right)=18-6x+3x(x+7)$ ist die Umformung zu $(9 - 3x)(x + 7) = 18 - 6x$.   1. Ist das ein guter Schritt? (Kreuze an) a. Der Schritt ist korrekt und er bringt viel b. Der Schritt ist korrekt, aber er bringt nicht viel c. Der Schritt ist falsch. 2. Begründe deine Antwort. | Option a. wird angekreuzt.  Begründung: Der Schritt ist korrekt, weil auf beiden Seiten korrekt subtrahiert und anschliessend auch korrekt ausgeklammert wurde. Die Lösung kann dann bestimmt werden, indem $x+7=1$ und $\left( 9-3x \right)=0$ gesetzt wird. |
| Konzeptuelles Wissen (*n* = 20) | 1. Lässt sich der Ausdruck $(x+7)(2x-5)$ umformen zu (ankreuzen) a. $x(2x - 5) + 7(2x - 5)$ b. $(x + 6)(2x - 5) + 2x - 5$ 2. Lässt sich die Gleichung $2x - 7 = 15 + \left( x - 1 \right)\left( 2x - 7 \right)$ umformen zu (ankreuzen) a. $0 = 15 + \left( x - 2 \right)\left( 2x - 7 \right)$ b. $0 = 15 + (x - 2)(x - 3) + (x - 2)(x - 4)$ 3. Wie viele (reelle) Lösungen hat die Gleichung $x^{2}=7$ (ankreuzen) a. Genau zwei Lösungen b. Genau eine Lösung c. Keine Lösung d. Das kann ich erst sagen, wenn ich sie gelöst habe. | 1. a. und b. sind angekreuzt 2. a. und b sind angekreuzt 3. a. ist angekreuzt |
|  | |  |

# Anhang B: Sequenzielle Modelle

Tab. B1: Sequenzielle Modelle für den Leistungszuwachs zwischen Pre- und Posttest (mit nur einem Prädiktor)

|  | Strategienutzung | | Strategiewissen | | Prozedurales Wissen | | Konzeptuelles Wissen | |
| --- | --- | --- | --- | --- | --- | --- | --- | --- |
|  | unstandardisiert (Standardfehler) | standardisiert | unstandardisiert (Standardfehler) | standardisiert | unstandardisiert (Standardfehler) | standardisiert | unstandardisiert (Standardfehler) | standardisiert |
| γ_00_ (Intercept) | 1.82 (0.48)*** | .56 | 1.37 (10.2) | ns | 3.59 (1.52)** | 1.79 | 3.18 (1.39)* | 2.18 |
| β_between_ (Pretestscore) | 1.42 (0.22)*** | .77 | 1.09 (0.06)*** | .93 | 1.04 (0.20)*** | .85 | 0.90 (0.15)*** | .88 |
| γ_01_ (Vergleichsprompts) | 0.40 (0.08)*** | .43 | 0.19 (0.11)* | .15 | 0.24 (0.08)*** | .42 | 0.07 (0.04)* | .16 |
| γ_00_ (Intercept) | 1.89 (0.40)*** | .58 | 1.38 (1.08)† | ns | 4.69 (1.23)*** | 2.34 | 3.01 (1.35)* | 2.06 |
| β_between_ (Pretestscore) | 1.25 (0.15)*** | .68 | 1.08 (0.07)*** | .92 | 0.89 (0.16)*** | .73 | 0.90 (0.15)*** | .88 |
| γ_02_ (metakognitive Prompts) | 0.05 (0.01)*** | .48 | 0.03 (0.01)† | ns | 0.02 (0.01)*** | .34 | 0.01 (0.00)*** | .29 |
| γ_00_ (Intercept) | 3.47 (0.70)*** | 1.06 | 2.69 (1.44)* | .62 | 5.42 (1.22)*** | 2.70 | 3.31 (1.15)** | 2.14 |
| β_between_ (Pretestscore) | 1.31 (0.19)*** | .71 | 1.08 (0.08)* | .93 | 0.81 (0.17)*** | .72 | 0.91 (0.13)*** | .89 |
| γ_03_ (neutrale Prompts) | -0.01 (0.01) | ns | -0.03 (0.02)* | -.13 | 0.00 (0.01) | ns | 0.01 (0.01) | ns |
| γ_00_ (Intercept) | 3.68 (0.67)*** | 1.12 | 3.20 (1.51)* | .74 | 5.75 (1.32)*** | 2.88 | 3.43 (1.44)** | 2.36 |
| β_between_ (Pretestscore) | 1.27 (0.18)*** | .67 | 1.05 (0.08)*** | .90 | 0.84 (0.17)*** | .69 | 0.90 (0.15)*** | .87 |
| γ_04_ (einschränkende Prompts) | -0.02 (0.02)† | ns | -0.03 (0.01)** | -.13 | -0.01 (0.01) | ns | 0.00 (0.01) | ns |
| **p* < .05, ***p* < .01, ****p* < .001, † *p* < .1 (einseitiger Hypothesentest), ns: nicht signifikant | | | | | | | | |

# Anhang C: Leistungsdaten

Tab. C1: Mittelwert (Standardabweichung) der Schülerleistungen aus der Studie von Rüede et al. 2023

|  | Pretest | Posttest | Follow-up Test |
| --- | --- | --- | --- |
| Strategienutzung (max. 20 Punkte) | | | |
| Kontrollgruppe | 1.28 (2.57) | 3.04 (3.62) | 3.12 (3.82) |
| EG_Vergl._ | 1.96 (3.16) | 6.95 (6.63) | 6.28 (6.61) |
| EG_Vergl.&AT_ | **2.09** (3.53) | 6.67 (6.41) | 5.55 (6.35) |
| Strategiewissen (max. 50 Punkte) | | | |
| Kontrollgruppe | 14.03 (5.46) | 16.36 (5.88) | 17.29 (6.78) |
| EG_Vergl._ | **17.11** (7.35) | 20.87 (7.90) | 21.65 (8.21) |
| EG_Vergl.&AT_ | **16.06** (6.11) | 19.68 (7.76) | 19.76 (7.75) |
| Prozedurales Wissen (max. 20 Punkte) | | | |
| Kontrollgruppe | 6.38 (2.90) | 9.84 (4.29) | 9.83 (4.53) |
| EG_Vergl._ | 6.83 (3.50) | 12.12 (4.19) | 11.12 (4.46) |
| EG_Vergl.&AT_ | **7.29** (3.81) | 12.28 (4.64) | 11.18 (4.60) |
| Konzeptuelles Wissen (max. 24 Punkte) | | | |
| Kontrollgruppe | 8.02 (3.44) | 10.28 (3.61) | 10.46 (3.56) |
| EG_Vergl._ | **9.55** (3.78) | 11.84 (3.86) | 12.18 (4.20) |
| EG_Vergl.&AT_ | **9.06** (3.45) | 12.04 (3.78) | 11.81 (4.27) |
| *Hinweis:* Fettgedruckte Mittelwerte zeigen einen signifikanten Unterschied zwischen der entsprechenden Experimentalgruppe und der Kontrollgruppe | | | |

# Anhang D: Effektstärken des Leistungszuwachses

Tab. D1: Effektstärken (Cohen‘s *dz*) des Leistungszuwachses zwischen Pre- und Posttest

| Skala | *M*_pre_ | *M*_post_ | *SD*_Diff_ | Cohen‘s *dz* |
| --- | --- | --- | --- | --- |
| Strategienutzung | 1.78 | 5.53 | 4.65 | 0.81 |
| Strategiewissen | 15.73 | 18.94 | 5.67 | 0.57 |
| Prozedurales Wissen | 6.85 | 11.38 | 4.33 | 1.05 |
| Konzeptuelles Wissen | 8.91 | 11.38 | 3.71 | 0.67 |

Tab. D2: Effektstärken (Cohen‘s *dz*) des Leistungszuwachses zwischen Pre- und Follow-up-Test

| Skala | *M*_pre_ | *M*_post_ | *SD*_Diff_ | Cohen‘s *dz* |
| --- | --- | --- | --- | --- |
| Strategienutzung | 1.78 | 4.97 | 4.35 | 0.73 |
| Strategiewissen | 15.73 | 19.55 | 5.84 | 0.65 |
| Prozedurales Wissen | 6.85 | 10.72 | 4.25 | 0.91 |
| Konzeptuelles Wissen | 8.91 | 11.47 | 3.95 | 0.65 |
